# QTL mapping for pre-harvest sprouting resistance in *japonica* rice varieties utilizing genome re-sequencing

**DOI:** 10.1007/s00438-020-01688-4

**Published:** 2020-05-26

**Authors:** Kyeong-Seong Cheon, Yong Jae Won, Young-Min Jeong, Youn-Young Lee, Do-Yu Kang, Jun Oh, Hyoja Oh, Song Lim Kim, Nyunhee Kim, Eungyeong Lee, In Sun Yoon, Inchan Choi, Jeongho Baek, Kyung-Hwan Kim, Hyun-Su Park, Hyeonso Ji

**Affiliations:** 1 Department of Agricultural Biotechnology, National Institute of Agricultural Sciences, Rural Development Administration (RDA), Jeonju, 54874 South Korea; 2 Cheorwon Branch, National Institute of Crop Science, Rural Development Administration (RDA), Cheorwon, 24010 South Korea; 3 Seed Industry Promotion Center, Foundation of Agri. Tech. Commercialization & Transfer (FACT), Gimje, 54324 South Korea; 4 Crop Breeding Division, National Institute of Crop Science, Rural Development Administration (RDA), Wanju, 55365 South Korea

**Keywords:** *Japonica* rice, Pre-harvest sprouting, Re-sequencing, Quantitative trait locus

## Abstract

Pre-harvest sprouting (PHS) leads to serious economic losses because of reductions in yield and quality. To analyze the quantitative trait loci (QTLs) for PHS resistance in *japonica* rice, PHS rates on panicles were measured in 160 recombinant inbred lines (RILs) derived from a cross between the temperate *japonica* varieties Odae (PHS resistant) and Unbong40 (PHS susceptible) under two different environmental conditions—field (summer) and greenhouse (winter) environments. Genome re-sequencing of the parental varieties detected 266,773 DNA polymorphisms including 248,255 single nucleotide polymorphisms and 18,518 insertions/deletions. We constructed a genetic map comprising 239 kompetitive allele-specific PCR and 49 cleaved amplified polymorphic sequence markers. In the field environment, two major QTLs, *qPHS-3*^*FD*^ and *qPHS-11*^*FD*^, were identified on chromosomes 3 and 11, respectively, whereas three major QTLs, *qPHS-3*^*GH*^, *qPHS-4*^*GH*^, and *qPHS-11*^*GH*^, were identified on chromosomes 3, 4, and 11, respectively, in the greenhouse environment. *qPHS-11*^*GH*^ and *qPHS-11*^*FD*^ had similar locations on chromosome 11, suggesting the existence of a gene conferring stable PHS resistance effects under different environmental conditions. The QTLs identified in this study can be used to improve the PHS resistance of *japonica* varieties, and they may improve our understanding of the genetic basis of PHS resistance.

**Electronic supplementary material:**

The online version of this article (10.1007/s00438-020-01688-4) contains supplementary material, which is available to authorized users.

## Introduction

Pre-harvest sprouting (PHS) of rice (*Oryza sativa* L.) is a serious economic issue causing significant reductions in grain quality and yield primarily in *japonica* rice-cultivating regions. PHS involves the germination of grains on matured panicles before harvest, and it is typically promoted by frequent rainfall and high temperatures during the grain ripening or harvest season (Tejakhod and Ellis 2018). PHS is closely associated with the degree of seed dormancy, which inhibits seed germination under optimal environmental conditions (Takahashi 1980). Seed dormancy is a complex agronomic trait that is influenced by environmental factors, such as temperature and humidity (Takahashi 1980; Basbouss-Serhal et al. 2016), endogenous hormones, such as abscisic acid (ABA; promotes dormancy), and gibberellic acid (promotes germination) (Graeber et al. 2012; Ye et al. 2015), and some biological tissues, such as the embryo, endosperm, and maternal tissues (Gu et al. 2015). Because high levels of dormancy in seeds can cause undesirable results, such as preventing post-harvest germination, the balance between seed dormancy and its release must be maintained to enhance the yield and quality of rice.

The degree of seed dormancy is affected by genetic factors controlling germination (Gu et al. 2003; Li and Foley 1997). Regarding PHS and seed dormancy in cultivated and wild rice, several quantitative trait loci (QTLs) have been reported in the progenies of crosses between *japonica* and *indica* varieties (Lin et al. 1998; Miura et al. 2001; Dong et al. 2003; Guo et al. 2004; Wan et al. 2005, 2006; Chen et al. 2006; Jiang et al. 2006; Gao et al. 2008; Ji et al. 2009; Xie et al. 2011; Marzougui et al. 2012; Li et al. 2013; Sasaki et al. 2013; Wang et al. 2014; Lee and Kwon 2015) between *indica* varieties (Li et al. 2011), between *japonica* varieties (Fujino et al. 2004; Hori et al. 2010), between weedy rice lines and cultivars (Gu et al. 2004, 2005; Ye et al. 2010; Subudhi et al. 2012), between upland and lowland rice varieties (Mizuno et al. 2018), and between a wild rice (*Oryza rufipogon)* line and a cultivar (Cai and Morishima 2000). These QTLs were distributed across all 12 chromosomes, and among them, a few genes have been identified via map-based cloning (Ye et al. 2015; Fujino et al. 2008; Sugimoto et al. 2010). *qSD1-2* on chromosome 1, which is associated with seed dormancy, was delimited to a 20-kb region containing *OsGA20ox2*, and the naturally occurring or induced loss-of-function mutations of this gene enhance seed dormancy (Ye et al. 2015). *qLTG3-1*, a QTL for low-temperature germinability on chromosome 3, was found to encode a protein of unknown function, and it is strongly expressed in the embryo during seed germination (Fujino et al. 2008). It was suggested that *qLTG3-1* causes tissue weakening of the epiblast covering the coleoptile and aleurone layers, resulting in reduced mechanical resistance to the growth potential of the coleoptile (Fujino et al. 2008). A rice QTL on chromosome 7, named *Seed dormancy 4* (*Sdr4*), was identified and revealed to substantially contribute to differences in seed dormancy between *japonica* and *indica* varieties. *Sdr4* expression is positively regulated by *OsVP1*, a global regulator of seed maturation; further, *Sdr4* positively regulates potential regulators of seed dormancy and represses the expression of post-germinative genes, suggesting that this gene acts as an intermediate regulator of dormancy in the seed maturation process (Sugimoto et al. 2010). More recently, 10 SNP loci were identified as significantly affecting PHS through re-sequencing 21 representative rice accessions (10 PHS resistant and 11 PHS susceptible), and a regression equation for evaluating PHS based on the genotypes of eight significant loci was constructed, which accounted for an *R*^2^ value of 0.401 in japonica rice (Lee et al. 2017). Better understanding of the complexity of genetic factors for PHS and seed dormancy is required to accelerate the development of PHS-resistant varieties via marker-assisted breeding.

Generally, most *japonica* rice varieties have weaker seed dormancy than *indica* rice varieties, although relatively high PHS resistance has been detected in several *japonica* rice varieties (Lee et al. 2016). Despite the detection in the relatively high PHS resistance and seed dormancy among *japonica* rice varieties, genetic analyses including QTL mapping have been limited in *japonica* rice varieties because of their low frequencies of polymorphisms in traditional markers, such as restriction-fragment length polymorphism (RFLP) and simple sequence repeats (Zhang et al. 1996; Mackill 1995). Recently, next-generation sequencing (NGS) technology has enabled the large-scale detection of sequence variations among *japonica* rice varieties (Hori et al. 2017; Jeong et al. 2015; Cheon et al. 2018) and has facilitated the development of high-throughput single nucleotide polymorphism (SNP) marker and QTL mapping in *japonica* varieties and populations. Among the high-throughput SNP markers, the kompetitive allele-specific PCR (KASP) marker system has the advantages of improved cost efficiency and time-effectiveness (Semagn et al. 2014; Yuan et al. 2014). Previously, we analyzed genome sequence data from 13 Korean *japonica* rice varieties and discovered 740,566 SNPs among these varieties. Based on this result, we developed 771 KASP markers for *japonica* rice varieties (Cheon et al. 2018, 2019).

In this study, KASP and cleaved amplified polymorphic sequence (CAPS) markers were used to construct a genetic map using 160 recombinant inbred lines (RILs) derived from a cross between the *japonica* rice varieties Odae (PHS-resistant) and Unbong40 (PHS susceptible). Five QTLs for PHS resistance were identified in this RIL population under two environmental conditions. The QTLs of *qPHS-11*^*GH*^ and *qPHS-11*^*FD*^ which were identified under greenhouse and field conditions, respectively, had similar locations on chromosome 11, suggesting the existence of a gene conferring stable PHS resistance effects under different environmental conditions, and will be useful in molecular breeding of PHS-resistant rice varieties.

## Results

### Phenotypic variations of PHS

**Fig. 1:**
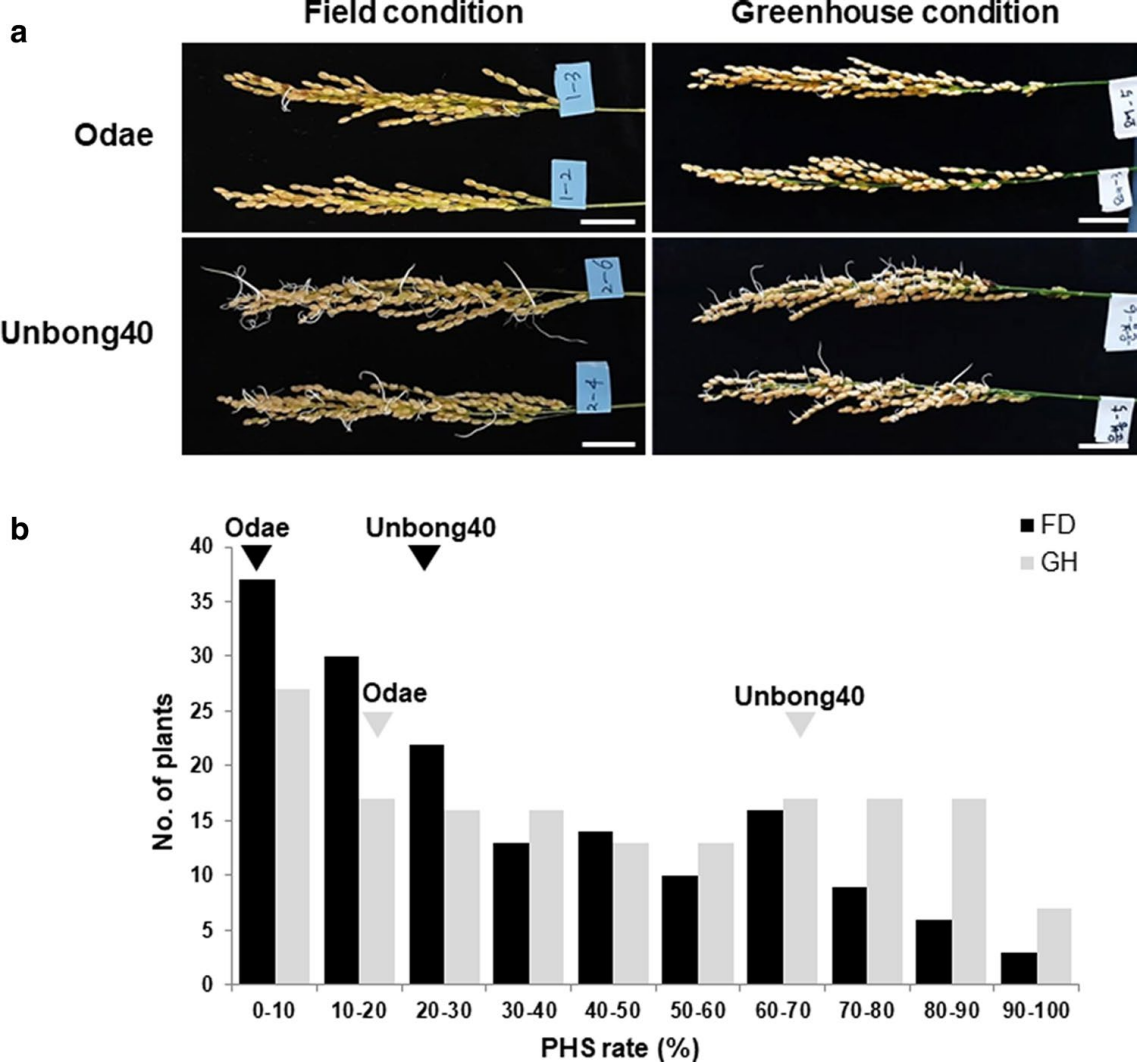
Phenotypic variations of pre-harvest sprouting (PHS) in the parental varieties under two environmental conditions. **a** PHS of parental varieties. Panicles were harvested 35 days after flowering and incubated for 7 days under 25 °C and 100% humidity. Each bar indicates 3 cm. **b** The distribution of the PHS rates of 160 recombinant inbred lines derived from a cross between Odae and Unbong40. FD and GH denote field and greenhouse conditions, respectively. Solid inversed triangles on the histogram indicate the PHS rates of the parental varieties Odae and Unbong40

We cultivated an F_9_ RIL population from a cross between Odae and Unbong40 in two environmental conditions (greenhouse in winter and field in summer) and measured PHS rates. The mean PHS rate of Unbong40 was higher than that of Odae in both environments (Fig. 1a), and the average PHS rates of Odae and Unbong40 were 1.5% and 36.4%, respectively, in the field and 19.5% and 69.3%, respectively, in the greenhouse (Fig. 1b). The mean PHS rate of the 160 RILs ranged continuously from 0 to 100% under both conditions, and a frequency of 0–10% class was the highest in both environmental conditions (Fig. 1b). The analysis of variance of the means of the PHS rates under both conditions was performed to test the significance of differences between lines and replications (Table 1). The differences between lines and replications were both highly significant.

**Table 1:** Analysis of variance of the pre-harvest sprouting rates of 160 recombinant inbred lines

Environmental conditions	Source	df*	Sum of square	Mean square	*F* value	Probability
Field	Line	159	420,034.22	2641.72	90.09	7.46E−282
	Replication	3	18,681.56	6227.19	212.36	1.81E−87
	Error	477	13,987.48	29.32		
Greenhouse	Line	159	530,578.80	3336.97	128.34	0
	Replication	3	18,363.88	6121.29	235.43	1.06E−93
	Error	477	12,402.16	26.00		

*df degree of freedom

### Re-sequencing analysis of parental varieties

The parental Odae and Unbong40 varieties were re-sequenced, and the obtained raw sequence data for the varieties were approximately 14.55 and 15.06 Gbp, respectively (Table 2). After quality trimming and read mapping onto the Nipponbare reference genome sequence (IRGSP-1.0, https://rapdb.dna.affrc.go.jp/dowmload/irgsp1.html), 106.07 × 10^6^ reads containing 10.37 Gbp were mapped with an average mapping depth of 27.28 × for the Odae variety, whereas 103.99 × 10^6^ reads containing 10.16 Gbp were mapped with a mapping depth of 27.22 × for the Unbong40 variety. Between Odae and Unbong40, 266,773 DNA polymorphisms including 248,255 SNPs and 18,518 insertions/deletions (indels) were detected (Table S1). The number of DNA polymorphisms detected varied greatly from chromosome to chromosome from a high of 82,593 on chromosome 8 to a low of 3267 on chromosome 5. Regions of high and low SNP density were distributed unevenly over all chromosomes (Fig. S1).

**Table 2:** Summary of the sequencing data of parental varieties

Varieties	Raw sequencing data		After quality trimming (Q20*)			After read mapping		
	No. of reads (× 10^6^)	Nucleotides (Gb)	No. of reads (× 10^6^)	Nucleotides (Gb)	Sequencing depth ( ×)	No. of reads (× 10^6^)	Nucleotides (Gb)	Average mapping depth ( ×)
‘Odae’	144.07	14.55	125.95	12.30	32.95	106.07	10.37	27.28
‘Unbong40’	149.13	15.06	130.15	12.70	34.04	103.99	10.16	27.22

*The “Q20” value indicates an accuracy of 99% for the base called

### Genetic map construction and QTL mapping

**Fig. 2:**
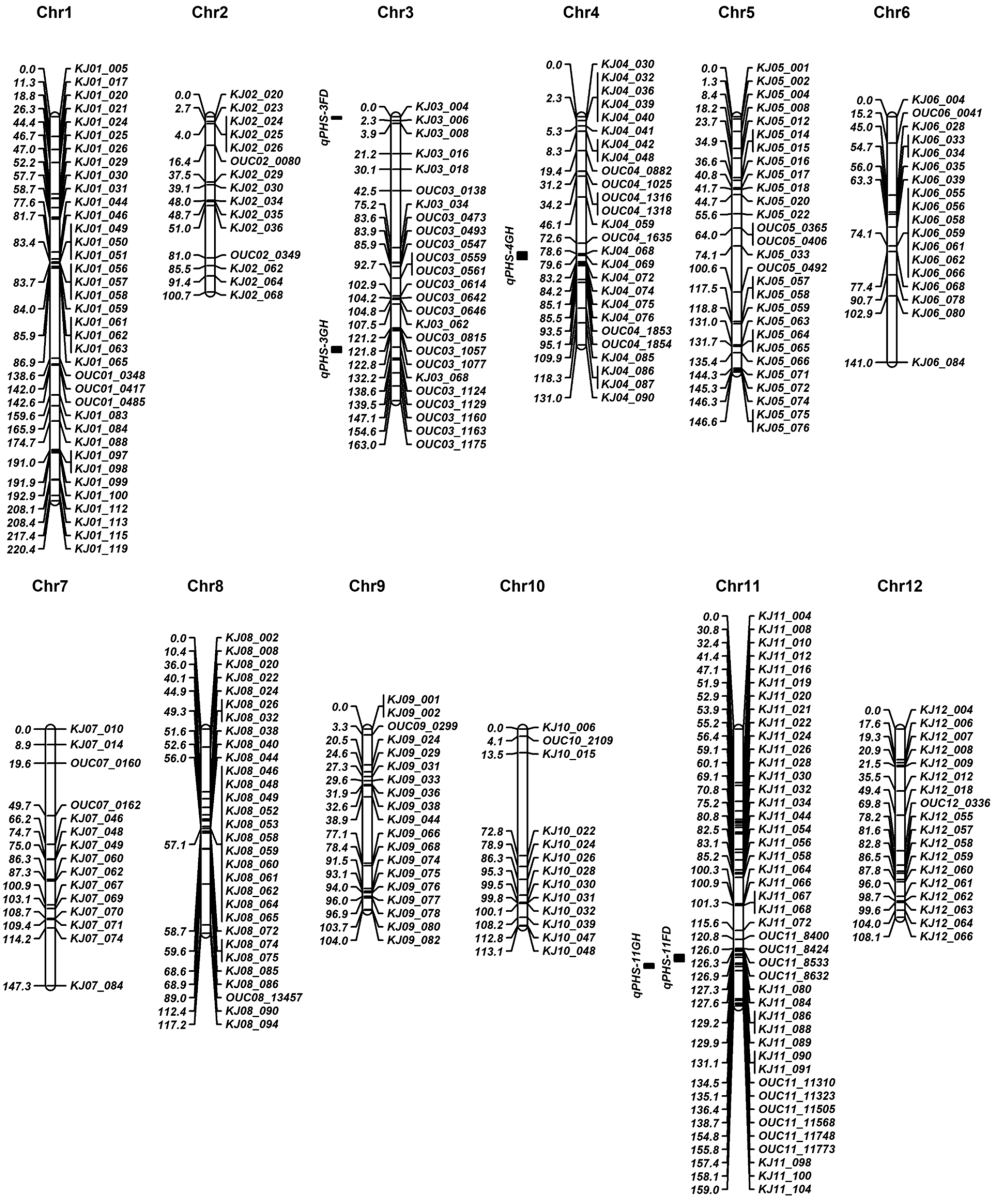
Construction of a genetic map using 239 kompetitive allele-specific PCR and 49 cleaved amplified polymorphic sequence markers with genotypes of 160 recombinant inbred line plants derived from a cross between the varieties Odae and Unbong40. The chromosome number is indicated at the top of each chromosome, the name of each marker is indicated on the right side of each chromosome, and the genetic distance of each marker from the first marker at the top of each chromosome is shown on the left side. Genetic distances, measured in cM, were calculated using the Kosambi function. The quantitative trait locus interval at 95% probability is indicated by the filled black box

Using 242 KASP markers selected from the previous studies (Cheon et al. 2018, 2019), we genotyped 160 RIL plants derived from a cross between Odae and Unbong40. Reliable genotypic data were obtained from 239 KASP markers with three markers producing unusable data owing to poor allelic discrimination. An initial genetic map comprising 239 KASP markers was constructed (Fig. S2). CAPS markers developed (Table S2) based on the detected SNPs between Odae and Unbong40 through the aforementioned re-sequencing analysis were used to genotype the RILs to fill in gaps. A final genetic map comprising 288 markers including 239 KASP and 49 CAPS markers was constructed (Fig. 2). The total length of this genetic map was 1651.5 cM, and the average interval between markers was 5.98 cM.

Using the genetic map and phenotypic data of 160 RILs from the Odae/Unbong40 cross, QTL mapping of the PHS rate was performed (Fig. S2). The QTL analysis with PHS field data revealed two major QTLs, *qPHS-3*^*FD*^ and *qPHS-11*^*FD*^, located at 0 cM on chromosome 3 and 129.91 cM on chromosome 11, respectively, with logarithm of the odds (LOD) scores of 13.86 and 13.96, respectively. The LOD threshold was calculated to be 6.7 through a 1000 × permutation with a probability level of 0.05 (Fig. S3). The additive effects of these QTLs were -13.27 and -13.10, respectively with *R*^*2*^ of 0.237 and 0.235, respectively, whereas Odae alleles decreased PHS rate. The closest markers of these QTLs were *KJ03_004* and *KJ11_086*, respectively, and the QTL intervals at 95% probability were 0–1.2 and 129.5–132.7 cM, respectively (Table 3). The *qPHS-3*^*FD*^ region was flanked by the markers of *KJ03_004* and *KJ03_006* (0–1.54 Mbp) on chromosome 3, whereas the *qPHS-11*^*FD*^ region was flanked by *KJ11_088* and *OUC11310* (22.89–24.03 Mbp) on chromosome 11 (Fig. S4). Moreover, the QTL analysis with greenhouse phenotype data of PHS identified three QTLs, namely *qPHS-3*^*GH*^, *qPHS-4*^*GH*^, and *qPHS-11*^*GH*^, which were located at 133.21 cM on chromosome 3, 78.61 cM on chromosome 4, and 135.21 cM on chromosome 11, respectively, with LOD scores of 18.1, 10.3, and 14.3, respectively. The LOD threshold was calculated to be 6.8 through a 1000 × permutation with a probability level of 0.05 (Fig. S3). The additive effects of these QTLs were − 14.02, 9.50, and − 11.52, respectively, with *R*^*2*^ of 0.222, 0.108, and 0.152, respectively. The Odae alleles of *qPHS-3*^*GH*^ and *qPHS-11*^*GH*^ decreased PHS rate, whereas that of *qPHS-4*^*GH*^ increased PHS rate. The closest markers of these QTL were *KJ03_068*, *KJ04_068*, and *OUC11_11310*, respectively, and the QTL intervals at 95% probability were 132.1–135.2, 129.5–132.7, and 134.7–137.3 cM, respectively (Table 3). The *qPHS-3*^*GH*^, *qPHS-4*^*GH*^, and *qPHS-11*^*GH*^ regions were flanked by the markers of *KJ03_068* and *OUC03_1124* (28.53–29.30 Mbp) on chromosome 3, *OUC04_1635* and *KJ04_072* (21.15–23.88 Mbp) on chromosome 4, and *OUC11_11310* and *OUC11_11568* (24.03–24.81 Mbp) on chromosome 11, respectively (Fig. S4).

**Table 3:** Quantitative trait loci associated with the pre-harvest sprouting rates of 160 recombinant inbred lines derived from a cross between the *Japonica* varieties Odae and Unbong40

Environmental condition	QTL name	Chr.	Location (cM)	LOD**	Closest marker	QTL interval*** (cM)	Additive effect	*R*^2^	References
Field	*qPHS-3*^*FD*^	3	0	12.33	*KJ03_004*	0–1.2	− 11.72	0.206	*qSdnj-3* (Wan et al. 2006) *qSD-3* (Miura et al. 2002) *qLTG3-1* (Fujino et al. 2004) *qLTG3-1* gene (Fujino et al. 2008)
	*qPHS-11*^*FD*^	11	130.91	12.28	*KJ11_090*	129.5–133.5	− 11.76	0.205	*qLTG-11* (Miura et al. 2001) *qSD-11* (Miura et al. 2002) *qLTG-11–1* and *-2* (Jiang et al. 2006) *qSD11*^*BR*^ (Subudhi et al. 2012) *qDOR-11–4, -5,* and *-6* (Cai and Morishima 2000)
Greenhouse	*qPHS-3*^*GH*^	3	133.21	18.12	*KJ03_068*	132.1–135.2	− 14.02	0.222	*qLTG-3–2* (Fujino et al. 2004)
	*qPHS-4*^*GH*^	4	78.61	10.34	*KJ04_068*	77.7–82.0	9.50	0.108	*qLTG-4–2* (Miura et al. 2001) *qSD*^*s*^*-4* (Gu et al. 2004) *qSD4* (Gu et al. 2005)
	*qPHS-11*^*GH*^	11	135.21	14.32	*OUC11_11310*	134.7–137.3	− 11.52	0.152	*qLTG-11* (Miura et al. 2001) *qSD-11* (Miura et al. 2002) *qLTG-11–1* and *-2* (Jiang et al. 2006) *qSD11*^*BR*^ (Subudhi et al. 2012) *qDOR-11–6* (Cai and Morishima 2000)

*Chr. Chromosome

**LOD logarithm of the odds

***Interval at 95% probability

### *qLTG3-1* genotype analysis

**Fig. 3:**
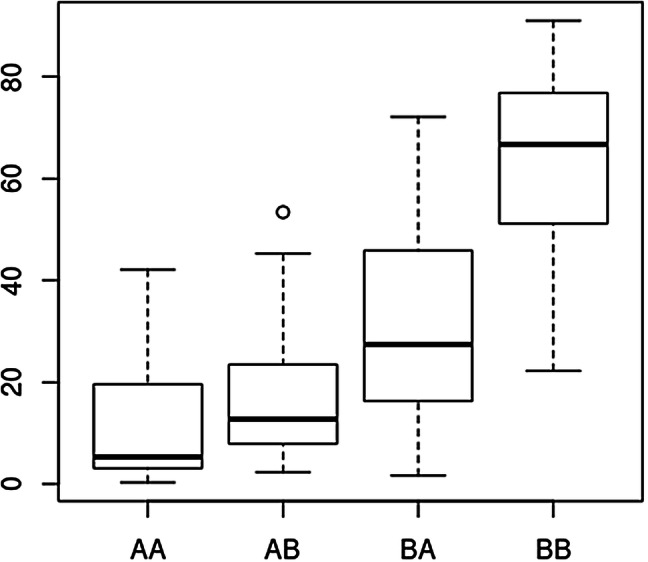
Boxplot showing the pre-harvest sprouting rate distribution of the four groups of recombinant inbred lines derived from a cross between Odae and Unbong40 classified by genotype combination of the two markers *qLTG3-1ind* and *KJ11_090*. The first letter in the genotype combination indicates the genotype of *qLTG3-1ind*, and the second letter indicates that of *KJ11_090*. ‘A’ denotes an Odae allele, and ‘B’ denotes an Unbong40 allele

The *qLTG3-1* gene (*Os03g0103300*) controlling low-temperature germinability is located in the 219,979–220,919-bp region on chromosome 3. Italica Livorno exhibiting greater low-temperature germinability has a functional allele of *qLTG3-1*, whereas Hayamasari exhibiting weaker low-temperature germinability has a loss-of-function allele (Fujino et al. 2008). It was proposed that the allelic difference in *qLTG3-1* between Nipponbare and Koshihikari is likely associated with differences in PHS resistance (Hori et al. 2010). Interestingly, *qLTG3-1* is located within the 0–1.5-Mb genomic region of *qPHS-3*^*FD*^ detected in the present study. To reveal the relationship between PHS phenotype and genotype of this QTL, we investigated the genomic sequences of the *qLTG3-1* genes in Odae and Unbong40 and found a 71-bp deletion in the *qLTG3-1* gene sequences in the Odae allele (Fig. S4a). This deletion has also been found in Koshihikari (Hori et al. 2010). This 71-bp indel of the *qLTG3-1* gene was validated by PCR using the indel marker *qLTG3-1ind* (Fig. S5). Under the field cultivation condition, only two major QTLs, namely *qPHS-3*^*FD*^ and *qPHS-11*^*FD*^, were found, indicating that these QTLs might be useful for discriminating PHS-resistant and PHS-susceptible plants in field conditions. We classified the RILs into four classes according to the genotype combinations of the two markers for these QTLs (*qLTG3-1ind* and *KJ11_090*) and investigated the PHS rate distributions in each class. The group with Odae alleles at both QTLs displayed the lowest PHS rate, whereas the group with Unbong40 alleles at these loci had the highest PHS rate (Fig. 3). This indicates that selecting the Odae genotypes at these two QTLs may enable the selection of PHS-resistant plants under field conditions. In addition, we analyzed genotypes of the two markers with 29 Korean japonica rice varieties known to be resistant or susceptible to PHS (Kang et al. 2018; Ko et al. 2005; Park 2009; Park and Kim 2009). Among 15 PHS-resistant varieties, six varieties had Odae alleles at both markers, seven had an Odae allele and an Unbong40 allele, and two had Unbong40 alleles at both markers. Among 14 PHS-susceptible varieties, nine varieties had Unbong40 alleles at both markers, two had an Odae allele and an Unbong40 allele, and two had Odae alleles at both markers (Table S3). Therefore, PHS responses of 15 out of 29 varieties coincided with the genotype combinations of the two markers. This indicates that the genotype combination of these markers might considerably correlate with PHS response of japonica rice varieties and that there are other genes regulating PHS response in these japonica rice varieties.

### Analysis of candidate genes for the QTL on chromosome 11

Because *qPHS-11*^*FD*^ (22.89–24.03 Mbp) and *qPHS-11*^*GH*^ (24.03–24.81 Mbp) were detected in similar regions on chromosome 11, a common gene with a highly stable effect under extremely different environmental conditions might underlie these two QTLs. To identify candidate genes for these QTLs, annotations of DNA polymorphisms between Odae and Unbong40 were investigated in the 22.0–25.0-Mbp region on chromosome 11. In this region which harbors 155 genes, 2493 DNA polymorphisms including 2330 SNPs and 163 indels were detected. Based on the gene description information of the Nipponbare reference genome annotation database, we searched for genes potentially associated with seed germination, seed dormancy, or PHS among these genes. In this manner, 47 genes were selected. Among these genes, 34 carry non-synonymous SNPs (nsSNPs) and are regarded as potential candidate genes for PHS resistance (Table S4), whereas the remaining 13 genes are not considered likely candidates because their SNPs were located in untranslated regions (UTRs) or introns or they were synonymous SNPs (sySNPs) in protein coding sequences.

## Discussion

PHS is a quantitative trait, and many factors can contribute to PHS resistance, including seed dormancy. Environmental factors, such as temperature and moisture during the ripening period, also can affect the development of PHS. Moreover, seed dormancy is strongly affected by environmental conditions during seed development (Godinez-Palma et al. 2013), and the broad-sense heritability of seed germination on the day of harvest for the trait was low (Gu et al. 2015). Therefore, repeated experiments under different conditions are critical for increasing the accuracy of PHS resistance estimation. To identify QTLs highly related to the genetic control of PHS resistance, we performed cultivation under two different environmental conditions using the same RIL population derived from a cross between Odae and Unbong40. The differences between the phenotypes of parental varieties and the RIL population under two environmental conditions might be attributable to genotype–environment interactions considering the different conditions during the ripening periods between the field and greenhouse. For example, the average daily mean temperature varied over the range of 19.6–30.4 °C during the period from flowering to harvest in the field condition, whereas it remained relatively constant over the range of 25.4–26.8 °C in the greenhouse condition (Fig. S6). Moreover, natural rainfall affected RILs in the field condition but not the greenhouse condition. These differences might be major factors for detecting different QTLs under the two conditions.

The reported QTLs for PHS, seed dormancy, and low-temperature germinability were co-located with detected QTLs in this study. On chromosome 3, the *qPHS-3*^*FD*^ region (0–1.5 Mb) overlapped with QTLs that were previously detected in different backgrounds. *qSD-3* was detected at the marker interval *C515–C25* using 98 backcrossed inbred lines (BILs) derived from a cross between the *japonica* variety Nipponbare and *indica* variety Kasalath (Miura et al. 2002). *qSdnj-3* was detected at the marker interval *RM132–RM231* using a BC_1_ population derived from Nanjing35 (*japonica*)/N22 (*indica*)//Nanjing35 (Wan et al. 2006). *qLTG-3–1* for low-temperature germinability was located in the marker interval *GBR3001–GBR3002* using 122 BILs derived from a cross between the *japonica* varieties Italica Livorno and Hayamasari (Fujino et al. 2004), and the low-temperature germinability gene *qLTG3-1* was identified at the QTL (Fujino et al. 2008). Another QTL on chromosome 3, the *qPHS-3*^*GH*^ region (28.53–29.30 Mb), co-localized with *qLTG-3–2* detected in the marker interval *RM3436–RM2593* with the BILs from a cross between Italica Livorno and Hayamasari (Fujino et al. 2004). Regarding the QTL detected on chromosome 4, the *qPHS-4*^*GH*^ region (22.89–24.03 Mb) was co-located with three previously reported QTLs. *qSD4* for seed dormancy was mapped to the marker interval *RM564A–RM252* using 204 BC_1_ individual plants derived from crosses between the weedy rice line SS18-2 and *indica* type breeding line EM93-1 (EM93-1//EM93-1/SS18-2) (Gu et al. 2005). *qLTG-4–2* for low-temperature germinability was detected in the region *R93–C513* using 98 BILs derived from a cross between Nipponbare and Kasalath. The effect of the Kasalath allele on *qLTG-4–2*, which suppressed germination, was temporary, disappearing after 7–8 months of storage, suggesting that the lack of suppression of germination was attributable to the breaking of a residual effect of dormancy and that *qLTG-4–2* might control seed dormancy (Miura et al. 2001). On chromosome 11, *qSD-11* was detected in the marker interval *S2260–G376* using 98 BILs derived from a cross between Nipponbare and Kasalath (Miura et al. 2002). *qLTG-11–1* and *qLTG-11–2* at the marker interval *RM202–RM254* for low-temperature germinability were detected on chromosome 11 using 148 F_2_ population derived from a cross between the *japonica* variety USSR5 and *indica* variety N22 (Jiang et al. 2006). *qSD11*^*BR*^ in the marker interval *RM206–RM254* was detected from RILs derived from a cross between the Bengal variety and weedy rice line PSRR-1 (Subudhi et al. 2012). These reported QTLs overlapped with both the *qPHS-11*^*FD*^ (22.89–24.03 Mb) and *qPHS-11*^*GH*^ (24.03–24.81 Mb) regions. In addition, the *qPHS-11*^*FD*^ region overlapped with *qDOR-11–4*, *qDOR-11–5*, and *qDOR-11–6* located at the marker intervals *CDO365–C6a*, *R1465–RG1109*, and *RG1109–RZ536*, respectively, which were identified using 125 RILs derived from a cross between the *indica* variety Pei-kuh and wild rice line W1944 (*O. rufipogon* Griff.) (Cai and Morishima 2000). In contrast, *qPHS-11*^*GH*^ only co-located with *qDOR-11–6*. Hori et al. (2010) reported that a QTL including the *qLTG3-1* gene on chromosome 3 was detected with BILs of Nipponbare/Koshihikari//Nipponbare and chromosome segment substitution lines derived from crosses between Nipponbare and Koshihikari, and the level of germinability under low temperature was strongly correlated with the level of PHS resistance. Iwata and Fujino (2010) reported that *qLTG3-1* and *qLTG11* were effective for controlling low-temperature germinability in the Hoshinoyume and Koshihikari backgrounds, whereas only *qLTG3-1* was effective in the Hayamasari background. Moreover, the genotypes of the *qLTG3-1ind* marker in the *qPHS-3*^*FD*^ region and *KJ11_090* marker in the *qPHS-11*^*FD*^ region largely corresponded with the PHS rates of RILs in field environment. From these findings, it is expected that *qPHS-3*^*FD*^ and *qPHS-11*^*FD*^ play important roles in PHS resistance in *japonica* rice and that the *qLTG3-1ind* and *KJ11_090* markers, which are most tightly linked with *qPHS-3*^*FD*^ and *qPHS-11*^*FD*^, will have utility in marker-assisted breeding for reduced PHS in *japonica* rice varieties.

Subsequently, because *qPHS-11*^*FD*^ and *qPHS-11*^*GH*^ were located in similar regions and these two QTLs overlapped with most of the reported QTL regions on chromosome 11 in different rice varieties, we investigated gene annotation in the 22.0–25.0 Mb region containing *qPHS-11*^*FD*^ and *qPHS-11*^*GH*^. In total, 34 genes carrying nsSNPs were identified as possible candidate genes for *qPHS-11*^*FD*^ and *qPHS-11*^*GH*^ associated with PHS resistance. For example, *Os11g0600500* carried 11 nsSNPs in the protein coding sequences (CDSs) of the calcium-binding EF-hand domain containing protein. *Arabidopsis calmodulin-like 24 (CML24)* containing a calcium-binding EF-hand domain encodes a potential Ca^2+^ sensor that functions in response to ABA, and *CML24*-underexpressing transgenics are resistant to ABA-mediated inhibition of germination (Delk et al. 2005). The contents of and sensitivity to ABA were considered major variables in the regulation of dormancy and germination; specifically, ABA positively regulates dormancy induction and maintenance (Finch-Savage et al. 2017). In this context, *Os11g0586001* with three nsSNPs and one indel in the CDS might also be a strong candidate because it encodes a protein phosphatase 2C that plays a key role in the ABA signal transduction pathway and that can positively regulate seed germination (Bhatnagar et al. 2017). In addition, *Os11g0587600* can be a strong candidate because it encodes an ABC transporter that may be required for fatty acid transport during suberin deposition in the seed coat, which is necessary for seed dormancy (Fedi et al. 2017). Moreover, *Os11g0622800* can be another strong candidate because two significant SNPs associated with PHS in 80 *japonica* accessions, Chr11_24303075 and Chr11_24307321 (Lee et al. 2017), were located in this gene, and were also polymorphic between Odae and Unbong40.

Further fine mapping of the QTL region including both *qPHS-11*^*FD*^ and *qPHS-11*^*GH*^ on chromosome 11 and functional analysis of candidate genes using transgenic plants are required for the exact identification of genes and development of selection markers within these QTL regions.

## Materials and methods

### Plant growing and samplings

In total, 160 F_9_ RILs derived from a cross between Odae and Unbong40, which have early heading dates among *japonica* rice varieties, were cultivated in the National Institute of Agricultural Sciences of the Rural Development Administration (Jeonju, Republic of Korea) in the 2018–2019 season. The RILs were grown during winter in a greenhouse with maximum/minimum temperatures of 32 °C/22 °C and light/dark lengths of 14 h/10 h. Seeds of the two parental varieties and 160 F_9_ RILs were sown in early December 2018, and these seedlings were transplanted at three seedlings per growth pot of 150-mm diameter and 125-mm height (two pots per line) in late December 2018. During the cultivation of seedlings, a main culm was left in each seedling, and all tillers were removed to keep good growth under dense planting condition. In the field, the seeds of two parental varieties and 160 F_9_ RILs were sown in mid-May 2019, and these seedlings were transplanted at 20 seedlings in a row per line and 30 cm × 15 cm space at early June 2019. The tillers of plants in the field were not removed. For genotyping, genomic DNA from more than 10 seedlings per line was extracted using a Biomedic Plant gDNA Extraction Kit (Biomedic, Bucheon, Korea).

### Evaluation of PHS rates

PHS rates were evaluated according to the method by Kang et al. (2018) with slight modifications. The heading panicles were tagged by wrapping their culms with color tape. Their heading dates were recorded on the tape and data sheet. Six panicles per each line from six plants (one panicle from one plant) were harvested at approximately 35 days after heading. Harvested panicles were put on paper towels on stainless steel trays of 465 × 365 × 35-mm size. Tap water was added to the tray to slightly immerse panicles, and the panicles were covered with paper towels. The tray was covered with plastic wrap to prevent water loss through evaporation and put in an incubator at 25 °C. After 7 days of incubation, the numbers of germinated seeds and not-germinated seeds per panicle were counted. Seeds with shoots just emerged from a break in the husk were regarded to have germinated. The PHS rate (%) was calculated as (germinated seeds /total filled seeds) × 100%. The average PHS rates of RILs were calculated from four panicles per line removing maximum and minimum PHS rate panicles and used for QTL analysis.

### Parental variety re-sequencing and SNP analysis

We extracted genomic DNA from Odae and Unbong40 seedlings using a DNeasy Plant mini kit (Qiagen, Hilden, Germany). Sequencing libraries were created according to the manufacturer’s protocols using a TruSeq DNA PCR-free kit (Illumina, Inc., San Diego, CA, USA). The libraries were paired-end sequenced using the HiSeq 2000 system (Illumina, Inc.). The raw readouts that were graded as high-quality using Phred quality values > Q20 were used to analyze genetic variations between the Odae and Unbong40 varieties. As a reference genome sequence of rice, the *Oryza sativa* L. cv. Nipponbare sequence (IRGSP-1.0, https://rapdb.dna.affrc.go.jp/dowmload/irgsp1.html) was used. We used the CLC Assembly Cell program (ver. 3.2.2, https://www.clcbio.com) mainly for read mapping and SNP detection. The generated reads were mapped on to the Nipponbare reference sequence using the clc-mapper command with the following parameters: alignment mode, local; similarity, 95%; gap cost, 3; deletion cost, 3; mismatch cost, 2; length fraction, 1.0; and repeat, ignore. DNA polymorphisms between each parental variety and Nipponbare were detected using the clc_find_variation command. Thus, DNA polymorphisms including SNPs and small indels up to 5 bp in length were detected. DNA polymorphisms between Odae and Unbong40 were analyzed using Python programs developed in-house using the detected DNA polymorphisms between Nipponbare and Odae and between Nipponbare and Unbong40. The detected DNA polymorphisms were annotated as genic and intergenic based on positional information obtained from the Rice Annotation Project Database (RAP-DB, https://rapdb.dna.arc.go.jp/index.html) using a Python program developed in-house. Further, DNA polymorphisms in genic regions were classified as CDSs, UTRs, non-coding exons, and introns, and those in CDSs were separated as sySNPs and nsSNPs on the basis of amino acid substitutions.

### Genetic map construction and QTL mapping

Overall, 160 F_9_ RILs derived from a cross between Odae and Unbong40 were genotyped using 242 KASP markers, which showed polymorphisms between parental varieties among 771 previously developed KASP markers (Cheon et al. 2018, 2019). After genetic map construction using KASP markers and primary detection of QTLs to determine the PHS rate, CAPS markers were developed on all chromosomes to fill gaps in the first genetic map on the basis of SNPs detected via the re-sequencing of Odae and Unbong40 sequences. Among the detected SNPs from Odae and Unbong40 sequences, those located in restriction enzyme sites were extracted, and CAPS markers were designed as follows: 500-bp sequences flanking the left and right of SNPs located in restriction enzyme sites were used to design PCR primers using the BatchPrimer3 1.0 primer design program (https://probes.pw.usda.gov/batchprimer3/). The designed CAPS markers were used to test parental polymorphisms. PCR products amplified using the primers and parental DNA were digested using restriction enzymes and separated via electrophoresis on 1.2% agarose gels. The selected CAPS markers were used to analyze the genotypes of RIL plants.

Based on the genotypes of 160 RIL plants, a genetic map was constructed using the MapDisto 1.7 program (Lorieux et al. 2012) together with the MapChart program (Voorrips et al. 2002), and the Kosambi function was used for mapping. QTL analysis was performed via composite interval mapping (CIM) using the Windows QTL Cartographer ver. 2.5 program (Basten et al. 1996). Through a 1000 × permutation with a probability level of 0.05, the LOD threshold was calculated. CIM was performed with the default condition of the Windows QTL Cartographer ver. 2.5 program.

### Analysis of the QTL closest to the *qLTG3-1* gene

To clarify sequence differences within the *qLTG3-1* gene, which is located within the same genomic region as the target QTL on chromosome 3, we assembled genomic sequence reads of Odae and Unbong40 using the clc_assembler command of the CLC Assembly Cell program. Among the produced contigs after assembly, the contigs containing the *qLTG3-1* gene were identified using BLAST. From these contigs, the *qLTG3-1* sequences of Odae and Unbong40 were extracted and compared. A primer set was designed to validate an indel found between Odae and Unbong40 in the *qLTG3-1* gene (Fig. S4a). The developed indel marker was named *qLTG3-1ind* and was used to genotype160 RILs.

### Analysis of candidate genes on chromosome 11

The physical intervals of QTL regions including *qPHS-11*^*FD*^ and *qPHS-11*^*GH*^ on chromosome 11 were deduced from the positions of the left and right flanking markers of the QTL intervals at 95% probability. The DNA polymorphisms located in this target interval were extracted. The polymorphisms located in genic regions were selected according to the annotation of DNA polymorphisms. The gene description information from RAP-DB for the genes harboring DNA polymorphisms between Odae and Unbong40 were used as queries together with the terms “seed germination” or “seed dormancy” or “pre-harvest sprouting” to search related literature in the PubMed database (https://www.ncbi.nlm.nih.gov/pubmed). Through this strategy, the genes with DNA polymorphisms and molecular functions that are possibly related with PHS resistance were selected. Among these genes, those with nsSNPs or indels in CDSs were selected as final candidate genes, whereas those with sySNPs or DNA polymorphisms in introns and UTRs were excluded.

## Electronic supplementary material

Below is the link to the electronic supplementary material.

Supplementary file1 (DOCX 1298 kb)

## References

[CR1] Basbouss-Serhal I, Leymarie J, Bailly C (2016). Fluctuation of *Arabidopsis* seed dormancy with relative humidity and temperature during dry storage. J Exp Bot.

[CR2] Basten CJ, Weir BS, Zeng Z-B (1996). QTL cartographer: a reference manual and tutorial for QTL mapping.

[CR100] Bhatnagar N, Min MK, Choi EH, Kim N, Moon SJ, Yoon I, Kwon T, Jung KH, Kim BG (2017). The protein phosphatase 2C clade A protein OsPP2C51 positively regulates seed germination by directly inactivating OsbZIP10. Plant Mol Biol.

[CR3] Cai H-W, Morishima H (2000). Genomic regions affecting seed shattering and seed dormancy in rice. Theor Appl Genet.

[CR4] Chen L, Lou Q-J, Sun Z-X, Xing Y-Z, Yu X-Q, Luo L-J (2006). QTL mapping of low temperature on germination rate of rice. Rice Sci.

[CR5] Chen HC, Cheng WH, Hong CY, Chang YS, Chang MC (2018). The transcription factor OsbHLH035 mediates seed germination and enables seedling recovery from salt stress through ABA-dependent and ABA-independent pathways, respectively. Rice.

[CR6] Cheon K-S, Baek J, Cho Y-I, Jeong Y-M, Lee Y-Y, Oh J, Won YJ, Kang D-Y, Oh H, Kim SL, Choi I, Yoon IS, Kim K-H, Han J-H, Ji H (2018). Single nucleotide polymorphism (SNP) discovery and kompetitive allele-specific PCR (KASP) marker development with Korean *japonica* rice varieties. Plant Breed Biotech.

[CR7] Cheon K-S, Jeong Y-M, Lee Y-Y, Oh J, Kang D-Y, Oh H, Kim SL, Kim N, Lee E, Baek J, Choi I, Kim K-H, Won YJ, Yoon IS, Cho Y-I, Han J-H, Ji H (2019). Kompetitive allele-specific PCR marker development and quantitative trait locus mapping for bakanae disease resistance in Korean *japonica* rice varieties. Plant Breed Biotech.

[CR102] Delk NA, Johnson KA, Chowdhury NI, Braam J (2005). CML24, regulated in expression by diverse stimuli, encodes a potential Ca^2+^ sensor that functions in responses to abscisic acid, daylength, and ion stress. Plant Physiol.

[CR8] Dong Y, Tsozuki E, Kamiunten H, Terao H, Lin D, Matsuo M, Zheng Y (2003). Identification of quantitative trait loci associated with pre-harvest sprouting resistance in rice (*Oryza sativa* L.). Field Crops Res.

[CR103] Fedi F, O'Neill CM, Menard G, Trick M, Dechirico S, Corbineau F, Bailly C, Eastmond PJ, Penfield S (2017). Awake1, an ABC-Type transporter, reveals an essential role for suberin in the control of seed dormancy. Plant Physiol.

[CR9] Finch-Savage WE, Footitt S (2017). Seed dormancy cycling and the regulation of dormancy mechanisms to time germination in variable field environments. J Exp Bot.

[CR10] Fujino K, Sekiguchi H, Sato T, Kiuchi H, Nonoue Y, Takeuchi Y, Ando T, Lin SY, Yano M (2004). Mapping of quantitative trait loci controlling low-temperature germinability in rice (*Oryza sativa* L.). Theor Appl Genet.

[CR11] Fujino K, Sekiguchi H, Matsuda Y, Sugimoto K, Ono K, Yano M (2008). Molecular identification of a major quantitative trait locus, *qLTG3–1*, controlling low-temperature germinability in rice. PNAS.

[CR12] Gao FY, Ren GJ, Lu XJ, Sun SX, Li HJ, Gao YM, Luo H, Yan WG, Zhang YZ (2008). QTL analysis for resistance to preharvest sprouting in rice (*Oryza sativa*). Plant Breed.

[CR107] Godinez-Palma SK, Garcia E, Sanchez MD, Rosas F, Vazquez-Ramos JM (2013). Complexes of D-type cyclins with CDKs during maize germination. J Exp Bot.

[CR13] Graeber K, Nakabayashi K, Miatton E, Leubner-Metzger G, Soppe WJ (2012). Molecular mechanisms of seed dormancy. Plant Cell Environ.

[CR14] Gu XY, Chen ZX, Foley ME (2003). Inheritance of seed dormancy in weedy rice. Crop Sci.

[CR15] Gu X-Y, Kianian SF, Foley ME (2004). Multiple loci and epistases control genetic variation for seed dormancy in weedy rice (*Oryza sativa*). Genetics.

[CR16] Gu X-Y, Kianian SF, Hareland GA, Hoffer BL, Foley ME (2005). Genetic analysis of adaptive syndromes interrelated with seed dormancy in weedy rice (*Oryza sativa*). Theor Appl Genet.

[CR17] Gu X-Y, Zhang J, Ye H, Zhang L, Feng J (2015). Genotyping of endosperms to determine seed dormancy genes regulating germination through embryonic, endospermic, or maternal tissues in rice. G3.

[CR18] Guo L, Zhu L, Xu Y, Zeng D, Wu P, Qian Q (2004). QTL analysis of seed dormancy in rice (*Oryza sativa* L.). Euphytica.

[CR19] Hori K, Sugimoto K, Nonoue Y, Ono N, Matsubara K, Yamanouchi U, Abe A, Takeuchi Y, Yano M (2010). Detection of quantitative trait loci controlling pre-harvest sprouting resistance by using backcrossed populations of *japonica* rice cultivars. Theor Appl Genet.

[CR20] Hori K, Yamamoto T, Yano M (2017). Genetic dissection of agronomically important traits in closely related temperate japonica rice cultivars. Breed Sci.

[CR21] Iwata N, Fujino K (2010). Genetic effects of major QTLs controlling low-temperature germinability in different genetic backgrounds in rice (*Oryza sativa* L.). Genome.

[CR23] Jeong I-S, Kim T-H, Lee S-B, Suh S-C, Ji H (2015). Genome-wide detection of DNA polymorphisms between two Korean *japonica* rice varieties. Plant Breed Biotech.

[CR24] Ji SL, Jiang L, Wang YH, Zhang WW, Liu X, Liu SJ, Chen LM, Zhai HQ, Wan JM (2009). Quantitative trait loci mapping and stability for low temperature germination ability of rice. Plant Breed.

[CR25] Jiang L, Liu S, Hou M, Tang J, Chen L, Zhai H, Wan J (2006). Analysis of QTLs for seed low temperature germinability and anoxia germinability in rice (*Oryza sativa* L.). Field Crops Res.

[CR26] Kang S, Shon J, Kim H-S, Kim S-J, Choi J-S, Park J-H, Yoon Y, Sim J, Yang W (2018). Analysis of genetic variation in pre-harvest sprouting at different cumulative temperatures after heading of rice. Korean J Crop Sci.

[CR27] Ko J-C, Kim B-K, Lee K-S, Choi W-Y, Choi H-R, Choi E-A, Yun S-J (2005). Varietal difference in enzyme activities during preharvest germination of rice. Korean J Crop Sci.

[CR28] Lee J, Kwon SW (2015). Analysis of quantitative trait loci associated with seed germination and coleoptile length under low temperature condition. J Crop Sci Biotech.

[CR29] Lee G-A, Jeon Y-A, Lee H-S, Hyun D-Y, Lee J-R, Lee M-C, Lee S-Y, Ma K-H, Koh H-J (2016). Variation in pre-harvest sprouting resistance, seed germination and changes in abscisic acid levels during grain development in diverse rice genetic resources. Plant Genet Resour.

[CR30] Lee G-A, Jeon Y-A, Lee H-S, Hyun D-Y, Lee J-R, Lee M-C, Lee S-Y, Ma K-H, Koh H-J (2017). New genetic loci associated with preharvest sprouting and its evaluation based on the model equation in rice. Front Plant Sci.

[CR31] Li B, Foley ME (1997). Genetic and molecular control of seed dormancy. Trends Plant Sci.

[CR32] Li W, Xu L, Bai X, Xing Y (2011). Quantitative trait loci for seed dormancy in rice. Euphytica.

[CR33] Li L, Liu X, Xie K, Wang Y, Liu F, Lin Q, Wang W, Yang C, Lu B, Liu S, Chen L, Jiang L, Wan J (2013). *qLTG-9*, a stable quantitative trait locus for low-temperature germination in rice (*Oryza sativa* L.). Theor Appl Genet.

[CR34] Lin SY, Sasaki T, Yano M (1998). Mapping quantitative trait loci controlling seed dormancy and heading date in rice, *Oryza sativa* L., using backcross inbred lines. Theor Appl Genet.

[CR35] Lorieux M (2012). MapDisto: fast and efficient computation of genetic linkage maps. Mol Breed.

[CR36] Mackill DJ (1995). Classifying japonica rice cultivars with RAPD markers. Crop Sci.

[CR37] Marzougui S, Sugimoto K, Yamanouchi U, Shimono M, Hoshino T, Hori K, Kobayashi M, Ishiyama K, Yano M (2012). Mapping and characterization of seed dormancy QTLs using chromosome segment substitution lines in rice. Theor Appl Genet.

[CR38] Miura K, Lin SY, Yano M, Nagamine T (2001). Mapping quantitative trait loci controlling low temperature germinability in rice (*Oryza sativa* L.). Breed Sci.

[CR39] Miura K, Lin SY, Yano M, Nagamine T (2002). Mapping quantitative trait loci controlling seed longevity in rice (*Oryza sativa* L.). Theor Appl Genet.

[CR40] Mizuno Y, Yamanouchi U, Hoshino T, Nonoue Y, Nagata K, Fukuoka S, Ando T, Yano M, Sugimoto K (2018). Genetic dissection of pre-harvest sprouting resistance in an upland rice cultivar. Breed Sci.

[CR42] Park HS (2009) Studies on panicle traits and preharvest sprouting in rice (*Oryza sativa* L.). Dissertation, University of Seoul

[CR43] Park J-S, Kim H-D (2009). Viviparous germination characteristics of rice varieties adaptable to central region of Korea. Korean J Crop Sci.

[CR44] Sasaki K, Kazama Y, Chae Y, Sato T (2013). Confirmation of novel quantitative trait loci for seed dormancy at different ripening stages in rice. Rice Sci.

[CR45] Semagn K, Babu R, Hearne S, Olsen M (2014). Single nucleotide polymorphism genotyping using kompetitive allele specific PCR (KASP): overview of the technology and its application in crop improvement. Mol Breed.

[CR46] Subudhi PK, Parco A, Singh PK, DeLeon T, Karan R, Biradar H, Cohn MA, Brar DS, Sasaki T (2012). Genetic architecture of seed dormancy in US weedy rice in different genetic backgrounds. Crop Sci.

[CR47] Sugimoto K, Takeuchi Y, Ebana K, Miyao A, Hirochika H, Hara N, Ishiyama K, Kobayashi M, Ban Y, Hattori T, Yano M (2010). Molecular cloning of *Sdr4*, a regulator involved in seed dormancy and domestication of rice. PNAS.

[CR48] Takahashi N (1980). Effect of environmental factors during seed formation on preharvest sprouting. Cereal Res Commun.

[CR49] Tejakhod S, Ellis RH (2018). Effect of simulated flooding during rice seed development and maturation on subsequent seed quality. Seed Sci Res.

[CR50] Voorrips RE (2002). MapChart: software for the graphical presentation of linkage maps and QTLs. J Hered.

[CR51] Wan JM, Cao YJ, Wang CM, Ikehashi H (2005). Quantitative trait loci associated with seed dormancy in rice. Crop Sci.

[CR52] Wan JM, Jiang L, Tang JY, Wang CM, Hou MY, Jing W, Zhang LX (2006). Genetic dissection of the seed dormancy trait in cultivated rice (*Oryza sativa* L.). Plant Sci.

[CR53] Wang L, Cheng J, Lai Y, Du W, Huang X, Wang Z, Zhang H (2014). Identification of QTLs with additive, epistatic and QTL × development interaction effects for seed dormancy in rice. Planta.

[CR54] Xie K, Jiang L, Yue B, Yang CY, Li LF, Liu X, Zhang L, Zhao ZG, Wan JM (2011). Identification of QTLs for seed dormancy in rice (*Oryza sativa* L.). Plant Breed.

[CR55] Ye H, Foley ME, Gu X-Y (2010). New seed dormancy loci detected from weedy rice-derived advanced populations with major QTL alleles removed from the background. Plant Sci.

[CR56] Ye H, Feng J, Zhang L, Zhang J, Mispan MS, Cao Z, Beighley DH, Yang J, Gu X-Y (2015). Map-based cloning of seed dormancy1-2 identified a gibberellin synthesis gene regulating the development of endosperm-imposed dormancy in rice. Plant Physiol.

[CR57] Yuan J, Wen Z, Gu C, Wang D (2014). Introduction of high throughput and cost effective SNP genotyping platforms in soybean. Plant Genetics Genomics Biotech.

[CR58] Zhang Q, Zhou ZQ, Yang GP, Xu CG, Liu KD, Saghai Maroof MA (1996). Molecular marker heterozygosity and hybrid performance in indica and japonica rice. Theor Appl Genet.

