# Supplementary material for: QTL mapping for pre-harvest sprouting resistance in japonica rice varieties utilizing genome re-sequencing
Source: Mol Genet Genomics. 2020 May 26;295(5):1129–40. doi: 10.1007/s00438-020-01688-4 (PMC7391406; doi:10.1007/s00438-020-01688-4)
Supplement: Supplementary file 1 — Supplementary file1 (DOCX 1298 kb) [file 438_2020_1688_MOESM1_ESM.docx]

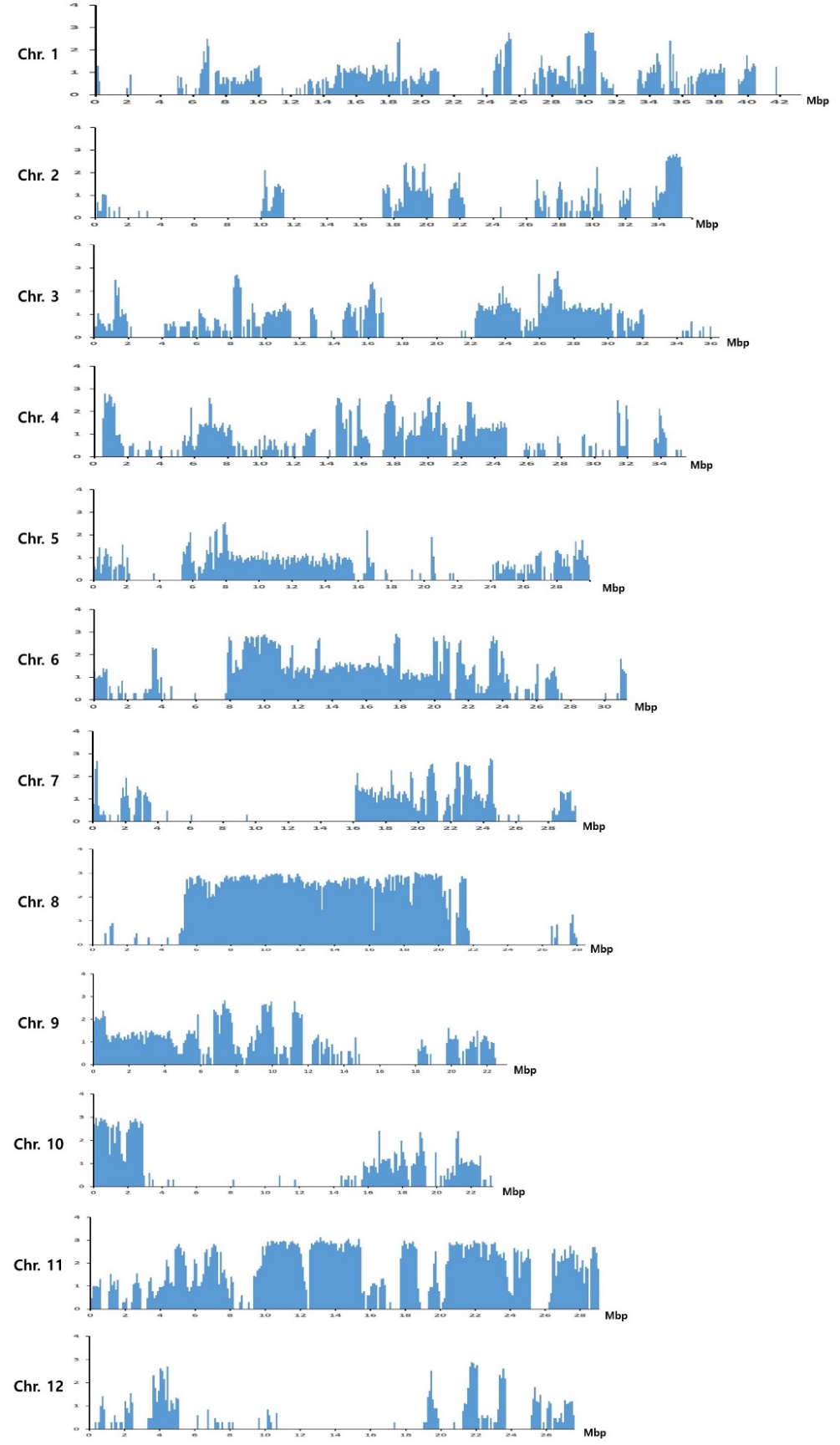
Fig. S1

Fig. S2


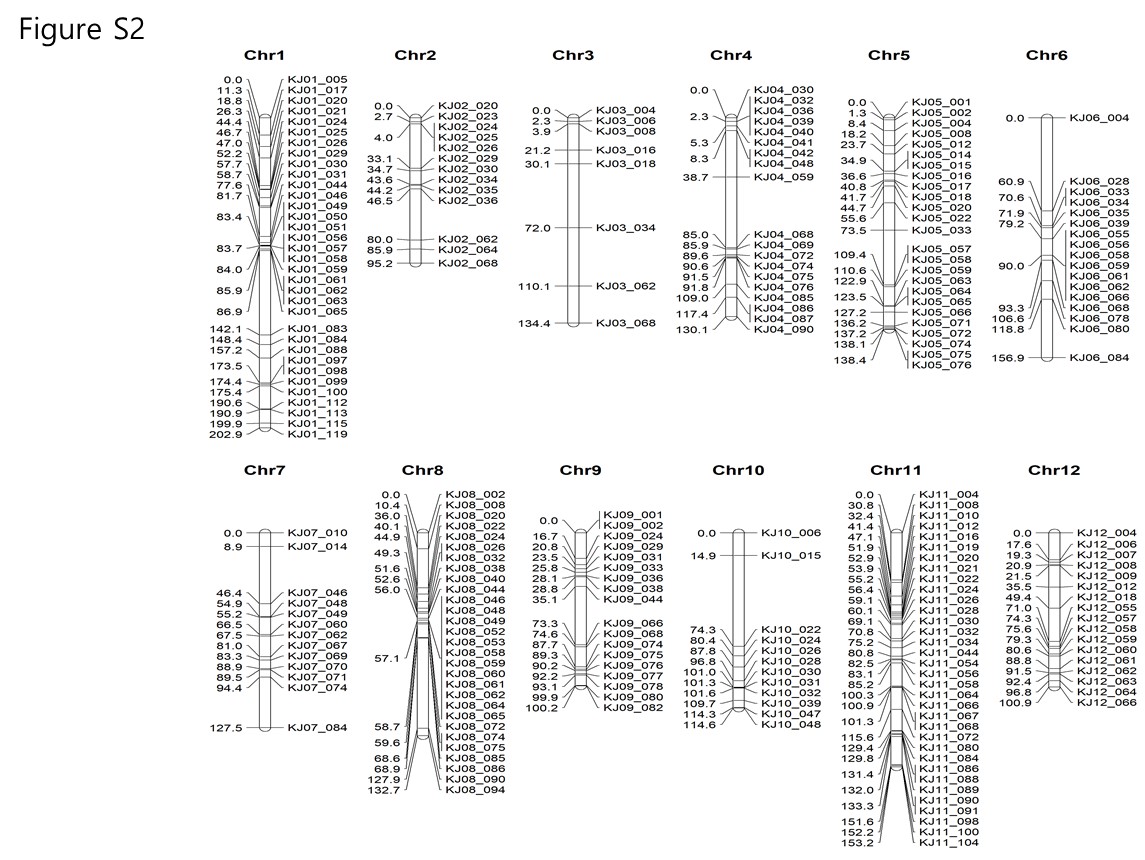


Fig. S3


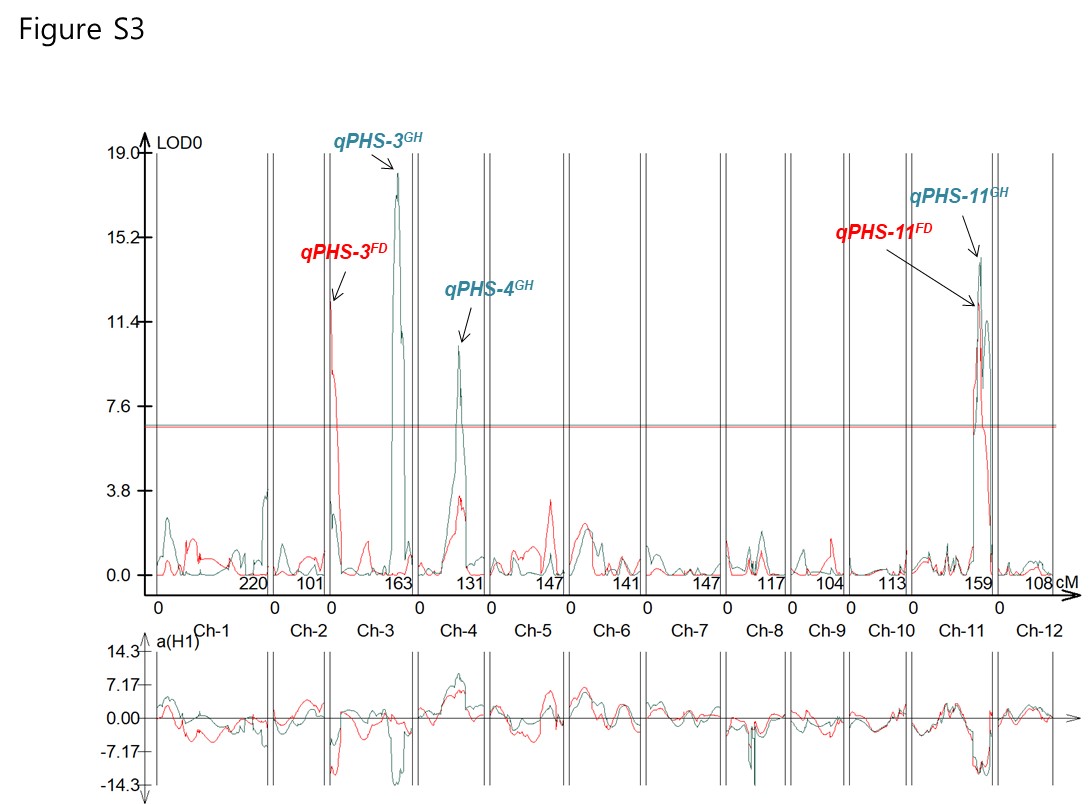


Fig. S4
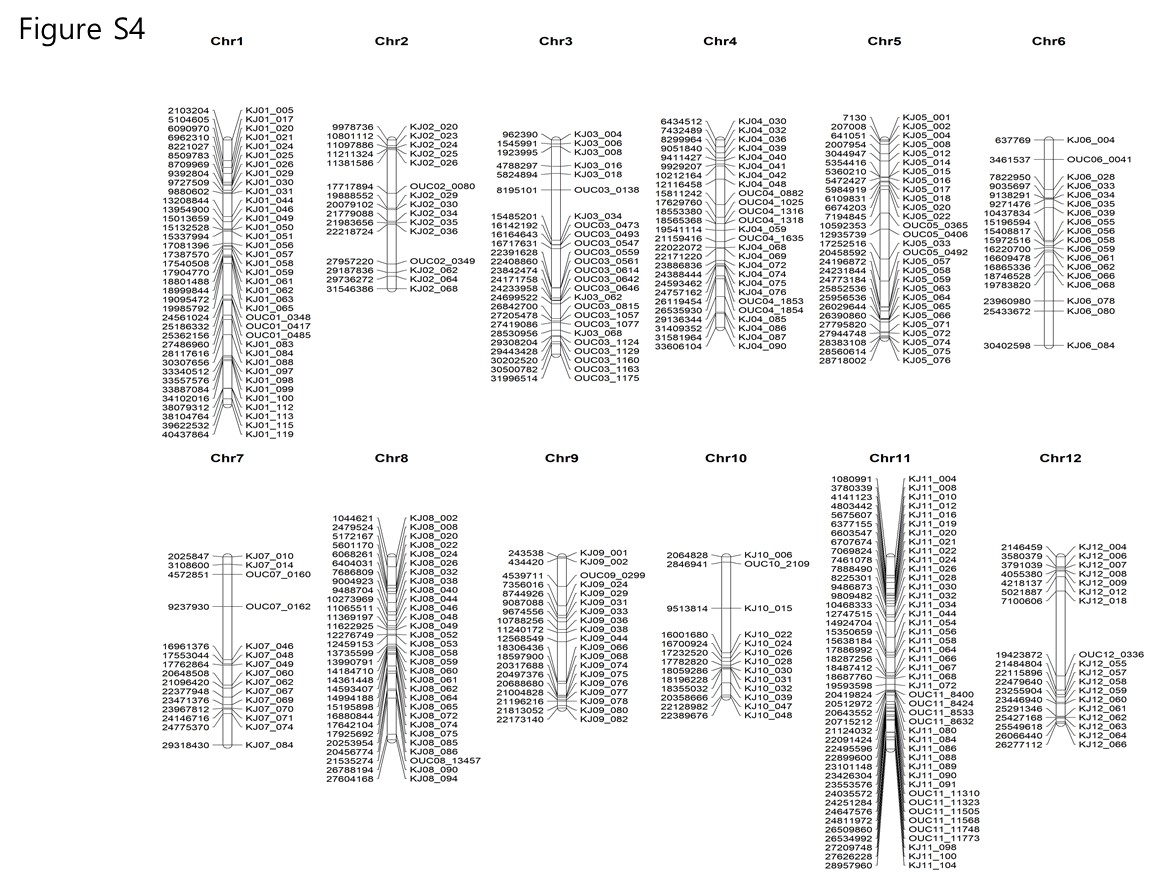


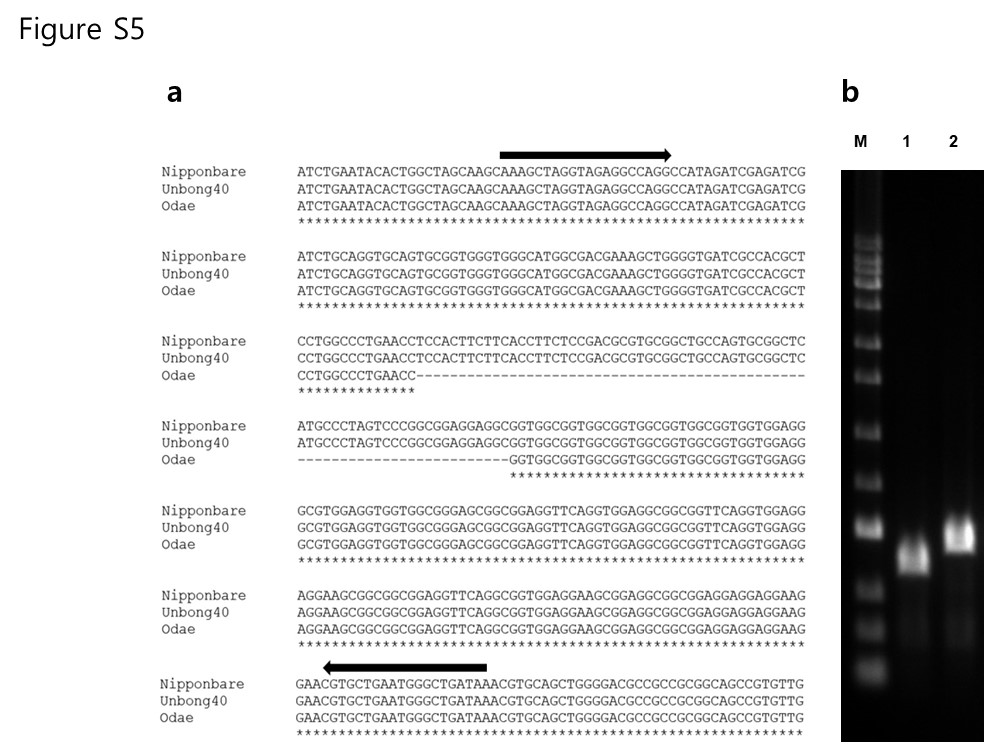
Fig. S5

Fig. S6


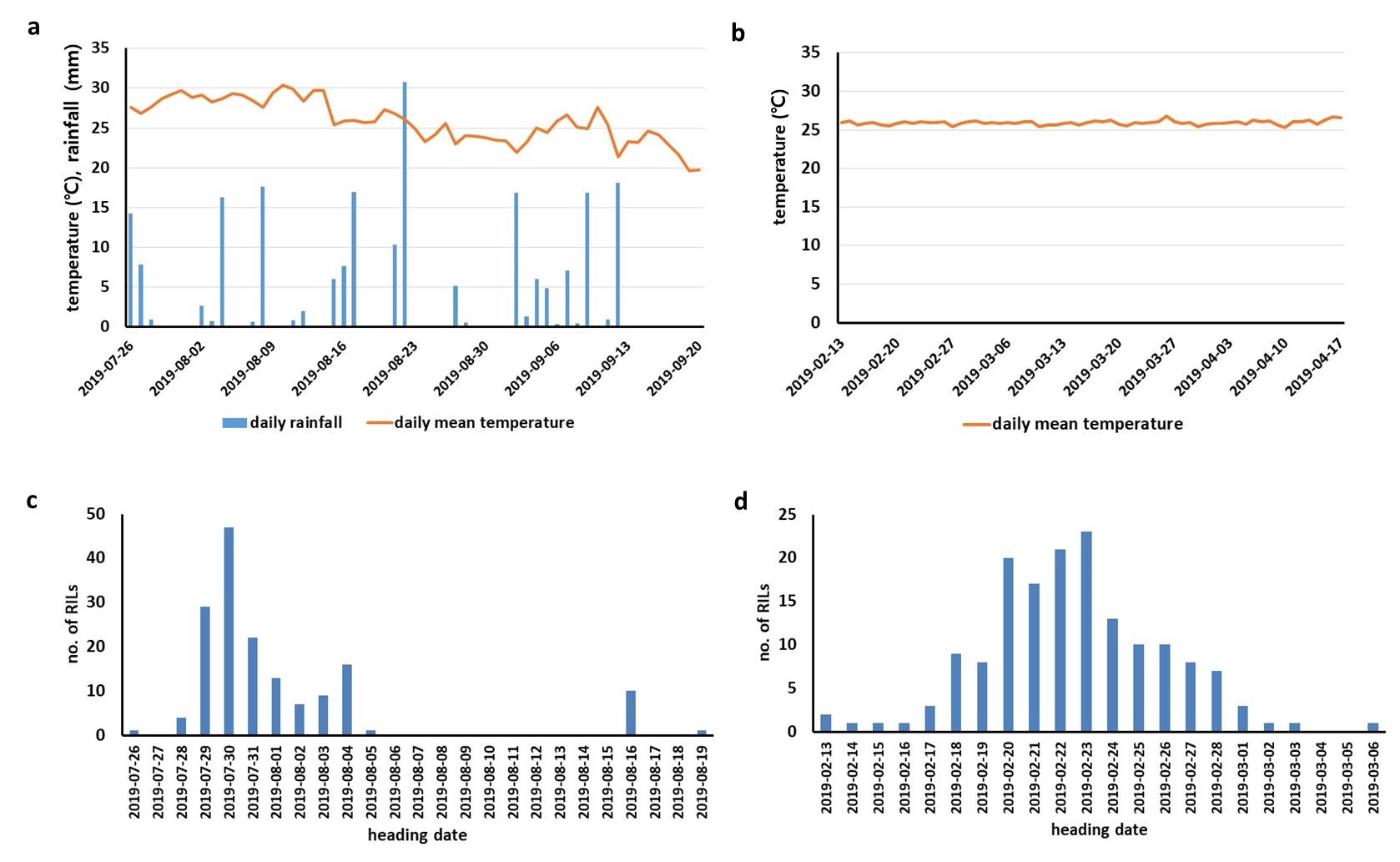


**Table S1.** Distribution of DNA polymorphisms between Odae and Unbong40 over all chromosomes

|  | SNP | InDel | Sum |
| --- | --- | --- | --- |
| Chr1 | 8,130 | 765 | 8,895 |
| Chr2 | 7,034 | 691 | 7,725 |
| Chr3 | 7,118 | 701 | 7,819 |
| Chr4 | 11,809 | 958 | 12,767 |
| Chr5 | 3,006 | 261 | 3,267 |
| Chr6 | 23,298 | 1,803 | 25,101 |
| Chr7 | 6,887 | 667 | 7,554 |
| Chr8 | 77,731 | 5,222 | 82,953 |
| Chr9 | 9,028 | 704 | 9,732 |
| Chr10 | 14,359 | 1,178 | 15,537 |
| Chr11 | 73,584 | 5,050 | 78,634 |
| Chr12 | 6,271 | 518 | 6,789 |
| total | 248,255 | 18,518 | 266,773 |

**Table S2.** List of cleaved amplified polymorphic sequence markers in this study

| **Chr^*^** | **Name** | **Position (bp)** | **Forward (5'-3')** | **Reverse (5'-3')** | **RE** |
| --- | --- | --- | --- | --- | --- |
| 1 | OUC01_0348 | 24561025 | TACGCCTGCCTCTACCTCAT | GATGAAGACGCGGTCGTAGA | *Bgl*II |
|  | OUC01_0417 | 25186333 | GACAAGGGCTCACTTTCGAC | GCCTCAGTTGTCTTCTGTTCG | *Bgl*II |
|  | OUC01_0485 | 25362156 | GCGAAGTGGTGTTTTTGGTT | GAGGGGTCACATCCCCTACT | *Eco*RI |
| 2 | OUC02_0080 | 17717894 | CGCCACCCTTTTTACTTTCA | TTCAAGGATCACCGTGAAGA | *Pvu*II |
|  | OUC02_0349 | 27957221 | AAGTGGTGGTTAGCGTCGTC | CACAGCGCACTACAGTAGCAG | *Pvu*II |
| 3 | OUC03_0138 | 8195101 | CTGCTCAGAATGGAATGCAC | AGCAAGGTGCCTCTGCTAAC | *Pvu*II |
|  | OUC03_0473 | 16142192 | TTGCGTGTAAATTGCGAGAC | CATTGCTACCCCATTTGCTT | *Eco*RV |
|  | OUC03_0493 | 16164643 | AAAGGGTGGTGATGAACCAG | TGAGTCTCACTCGGCTTTCA | *Bam*HI |
|  | OUC03_0547 | 16717631 | CATATTTTGGGAAATGGTCGAT | TGCAGCAGCTCTATCTTCAAA | *Alu*I |
|  | OUC03_0559 | 22391629 | TGTATCCAACCCGTCAATCC | AAACTTTCAGAGGGCGAAGG | *Mlu*I |
|  | OUC03_0561 | 22408860 | TCGGATTTGCCACATGATTA | AGGGGCCTTAGCCTCCTAGT | *Bgl*II |
|  | OUC03_0614 | 23842474 | TTGGTTTAATTCGGGATTGG | CAGTGTTTGGCTGAGACATGA | *Bam*HI |
|  | OUC03_0642 | 24171758 | GTCGTCGTGTGACCCAAACT | CCTTGTTGGGTCGAAACTGT | *Bam*HI |
|  | OUC03_0646 | 24233958 | GCTAGCGCACGCTAAACTAA | TCACTGCGGTCAGATATGGA | *Pst*I |
|  | OUC03_0815 | 26842701 | GTTGCATGCAGAAAGCTTGA | TCAGGGAGACCAGTTCGTTC | *Hin*dIII |
|  | OUC03_1057 | 27205478 | AATGTGCACCATGAGCCTTT | AGAAACCATGCCATTTGTCC | *Hin*dIII |
|  | OUC03_1077 | 27419086 | AAAAGGGAAGGAGGAGGAGA | AGCTGTTTGCCGAATAATCC | *Eco*RI |
|  | OUC03_1124 | 29308203 | GGCCTTGGGAAAACAATACA | GCAAGTTGTTTTGCGTCTGA | *Msp*I |
|  | OUC03_1129 | 29443429 | CGTACACCGCAAAATTCACA | CCAATTTTATCCGCGTGTTT | *Hin*fI |
|  | OUC03_1160 | 30202519 | CTCCCGGTATTGAGGGACTT | GGGCATATTTTGCTTCAACC | *Eco*RV |
|  | OUC03_1163 | 30500782 | CATGCAATCCACTGATCCTG | CATGTCTGCCATCAACTTGC | *Eco*RI |
|  | OUC03_1175 | 31996514 | CGCGAGGAAAAGGGAAAATA | AGAATGAACAGCACCAAGCA | *Mlu*I |
| 4 | OUC04_0882 | 15811242 | CCCCATCCCTCAGAACTATG | CTTGCGGTATGGCAAAAGTA | *Hin*dIII |
|  | OUC04_1025 | 17629760 | AGACGCCAAAATGATGAACC | GGCACTGTGAGAGGGAGAAA | *Bgl*II |
|  | OUC04_1316 | 18553381 | CATGTGGTGTTGCTGGACTT | CCAGGTCAGAGGAACTCAGG | *Bgl*II |
|  | OUC04_1318 | 18565369 | ATTATGCCCTTCGCTAATGC | GGCAGGCTCTAAACGGTGT | *Eco*RV |
|  | OUC04_1635 | 21159415 | TGTTCAATGTTGCCATTGATTT | TCAAACAGCATTACGTGCAA | *Pvu*II |
|  | OUC04_1853 | 26119454 | TGGCTCAACCACAACCACTA | GGCCAGAGATTCTGTTCCAG | *Msp*I |
|  | OUC04_1854 | 26535930 | GAAAGGAGACCACCGACAGA | CGACCTCGCCTCTTATCATC | *Sal*I |
| 5 | OUC05_0365 | 10592353 | CAGCTGGGTATGGTTGTTCA | AGTGGTTTGCATCGATGTTG | *Bam*HI |
|  | OUC05_0406 | 12935739 | CGAAACAAAACCCGTGTAGG | CGTTTCCTTCATCTGACCAAA | *Hin*dIII |
|  | OUC05_0492 | 20458593 | CTCCTCATTTCCCCTTTTCC | AAACCTCCACCAACCTCACA | *Msp*I |
| 6 | OUC06_0041 | 3461537 | CCTTCGCCGATCACTTAGAT | TTATGCTGCACCCGAGTATG | *Bgl*II |
| 7 | OUC07_0160 | 4572851 | CCTGCTTGGAAGGTCCATAA | TGGGAGTCTGGGAGAGATTG | *Msp*I |
|  | OUC07_0162 | 9237930 | CCAAGGACTAGGCCTCCAAT | CCGGCTATAACAACCGTGAC | *Cla*I |
| 8 | OUC08_13457 | 21535274 | GCTATAAAGACCGGCCACAA | TTATGGTGGACCCCACATTT | *Bam*HI |
| 9 | OUC09_0299 | 4539711 | CGGAAAGAGAAAAGGAAGCA | TCCCCTTCCAGGGATATCTT | *Eco*RI |
| 10 | OUC10_2109 | 2846941 | AATCGGTAACTTTCGGTGCT | CGAAGTAAGGGCAGGTGAAA | *Bam*HI |
| 11 | OUC11_8400 | 20419824 | GGCTGGAACGGTAGTGTCAT | ATGGGGAATGAGAATGTTGC | *Bam*HI |
|  | OUC11_8424 | 20512973 | CGTCCTCCTCTTGAAGCGTAT | GCCACGAAATTGTGAGTTGA | *Bam*HI |
|  | OUC11_8533 | 20643552 | CATTGCGTCTGACACTGCTT | AATGGCCAAAACCATGTAGC | *Hin*dIII |
|  | OUC11_8632 | 20715211 | TCACATTGGGGGCTCATATC | ACCGCTCCCTTTCTTTTCTC | *Eco*RI |
|  | OUC11_11310 | 24035571 | GTGCGGGCATATATCGTAGA | GCGCCATGTATAGTCCTGTG | *Hin*dIII |
|  | OUC11_11323 | 24251283 | GCGGCCTGAGACTAACACAT | ATGGTTCCCAAGAGACCTCA | *Hin*dIII |
|  | OUC11_11505 | 24647575 | TTGTGGATGCATGAACAGGT | TGAGTGTCACATCGGACGTT | *Eco*RI |
|  | OUC11_11568 | 24811972 | GATCTGGCCGTTCAATTCAT | TTGAGTGGCTTGGTTGAACA | *Bam*HI |
|  | OUC11_11748 | 26509861 | GATGAAAATGTGCCTGCTGA | TATCATTTTCTCCCCCACCA | *Eco*RI |
|  | OUC11_11773 | 26534991 | AGTCAAACCACACCCCAGTC | GGCTGATGCTTTTAGCTGGA | *Eco*RV |
| 12 | OUC12_0336 | 19423873 | AGGTTGCGCAAGACCTAAGA | CAACCGATGAGAGCACTAGC | *Eco*RI |

**^*^**Chr., Chromosome; RE, Restriction enzyme.

**Table S3.** Genotypes of Korean japonica rice varieties at *qLTG3-1ind* and *KJ11_090*

| No. | Variety | Genotype**^*^** | | PHS response**^**^** |
| --- | --- | --- | --- | --- |
|  |  | *qLTG3-1ind* | *KJ11_090* |  |
| 1 | Nunbora | A | A | R |
| 2 | Koshihikari | A | A | R |
| 3 | Hopyeong | A | A | R |
| 4 | Joun | A | A | R |
| 5 | Geumo | A | A | R |
| 6 | Ilpum | A | A | R |
| 7 | Jungsaenggold | A | B | R |
| 8 | Onnuri | A | B | R |
| 9 | Hwanggeumnuri | A | B | R |
| 10 | Heukhyang | A | B | R |
| 11 | Pungmi | A | B | R |
| 12 | Josaengheukchal | B | A | R |
| 13 | Hwaseong | B | A | R |
| 14 | Nampyeong | B | B | R |
| 15 | Mangeum | B | B | R |
| 16 | Boseokchal | B | B | S |
| 17 | Cheongho | B | B | S |
| 18 | Sinseonchal | B | B | S |
| 19 | Dongjin | B | B | S |
| 20 | Mihyang | B | B | S |
| 21 | Hwayeong | B | B | S |
| 22 | Daean | B | B | S |
| 23 | Hyangnam | B | B | S |
| 24 | Sindongjin | B | B | S |
| 25 | Ungwang | A | B | S |
| 26 | Dami | A | B | S |
| 27 | Juan | A | A | S |
| 28 | Jopyeong | A | A | S |
| 29 | Gopum | A | A | S |

**^*^**A, Odae genotype; B, Unbong40 genotype.

**^**^**PHS, pre-harvest sprouting; R, resistant; S, Susceptible.

**Table S4.** Identification of candidate genes in a region including *qPHS-11^FD^* and *qPHS-11^GH^*

| Gene ID | Description | Start (bp) | End (bp) | CDS^*^ | | |  | Intron | |  | 5' UTR^****^ | |  | 3' UTR | | Related literature^*****^ |
| --- | --- | --- | --- | --- | --- | --- | --- | --- | --- | --- | --- | --- | --- | --- | --- | --- |
|  |  |  |  | nsSNP^**^ | sySNP^***^ | indel |  | SNP | indel |  | SNP | indel |  | SNP | indel |  |
| Os11g0582700 | Cyclin-like F-box domain containing protein. | 22,025,760 | 22,029,748 | 8 | 1 | 0 |  | 3 | 0 |  | 1 | 0 |  | 4 | 0 | Godinez-Palma et al. 2013 |
| Os11g0583300 | Similar to F-box domain containing protein. | 22,046,396 | 22,046,842 | 2 | 0 | 0 |  | 0 | 0 |  | 0 | 0 |  | 0 | 0 | Majee et al. 2018 |
| Os11g0584100 | Cyclin-like F-box domain containing protein. | 22,068,104 | 22,070,310 | 2 | 1 | 0 |  | 1 | 0 |  | 0 | 0 |  | 2 | 0 | Godinez-Palma et al. 2013 |
| Os11g0585900 | Similar to ETO1-like protein 1 (Ethylene overproducer 1-like protein 1). | 22,150,902 | 22,155,445 | 2 | 3 | 0 |  | 7 | 1 |  | 0 | 0 |  | 1 | 0 | El-Maarouf-Bouteau et al. 2015 |
| Os11g0586001 | Putative protein phosphatase 2C 76. | 22,159,570 | 22,164,658 | 3 | 8 | 1 |  | 83 | 2 |  | 0 | 0 |  | 0 | 0 | Bhatnagar et al. 2017 |
| Os11g0586100 | Similar to protein kinase family protein | 22,167,971 | 22,173,473 | 4 | 10 | 0 |  | 54 | 9 |  | 0 | 0 |  | 8 | 1 | Zhou et al. 2015 |
| Os11g0586200 | Similar to Calmodulin. | 22,173,768 | 22,174,764 | 1 | 4 | 0 |  | 4 | 1 |  | 0 | 0 |  | 0 | 0 | Zhou et al. 2018 |

Table S4. continued.

| Gene ID | Description | Start (bp) | End (bp) | CDS^*^ | | |  | Intron | |  | 5' UTR^****^ | |  | 3' UTR | | Related literature^*****^ |
| --- | --- | --- | --- | --- | --- | --- | --- | --- | --- | --- | --- | --- | --- | --- | --- | --- |
|  |  |  |  | nsSNP^**^ | sySNP^***^ | indel |  | SNP | indel |  | SNP | indel |  | SNP | indel |  |
| Os11g0587000 | beta-carotene isomerase, Strigolactones biosynthesis | 22,221,628 | 22,226,596 | 2 | 0 | 0 |  | 16 | 4 |  | 0 | 0 |  | 0 | 0 | Fang et al. 2008 |
| Os11g0587600 | Similar to PDR-like ABC transporter. | 22,272,841 | 22,282,403 | 1 | 7 | 0 |  | 30 | 5 |  | 0 | 0 |  | 13 | 0 | Fedi et al. 2017 |
| Os11g0591100 | NAD(P)-binding domain containing protein. | 22,459,933 | 22,464,679 | 3 | 23 | 0 |  | 49 | 5 |  | 0 | 0 |  | 3 | 0 | Kirkensgaard et al. 2014 |
| Os11g0592300 | Glycosyl transferase, family 8 protein. | 22,514,191 | 22,516,597 | 3 | 0 | 0 |  | 1 | 0 |  | 0 | 0 |  | 0 | 0 | Liu et al. 2015 |
| Os11g0592900 | Thioredoxin domain 2 containing protein. | 22,566,058 | 22,568,588 | 3 | 1 | 0 |  | 9 | 0 |  | 1 | 0 |  | 2 | 0 | Ortiz-Espin et al. 2017 |
| Os11g0593500 | Cyclin-like F-box domain containing protein. | 22,604,874 | 22,606,587 | 6 | 4 | 1 |  | 0 | 0 |  | 1 | 0 |  | 0 | 1 | Godinez-Palma et al. 2013 |
| Os11g0593600 | Cyclin-like F-box domain containing protein. | 22,610,993 | 22,612,520 | 8 | 8 | 0 |  | 0 | 0 |  | 0 | 0 |  | 1 | 0 | Godinez-Palma et al. 2013 |
| Os11g0593700 | Cyclin-like F-box domain containing protein. | 22,614,877 | 22,616,875 | 7 | 8 | 0 |  | 0 | 0 |  | 1 | 0 |  | 2 | 0 | Godinez-Palma et al. 2013 |

Table S4. continued.

| Gene ID | Description | Start (bp) | End (bp) | CDS^*^ | | |  | Intron | |  | 5' UTR^****^ | |  | 3' UTR | | Related literature^*****^ |
| --- | --- | --- | --- | --- | --- | --- | --- | --- | --- | --- | --- | --- | --- | --- | --- | --- |
|  |  |  |  | nsSNP^**^ | sySNP^***^ | indel |  | SNP | indel |  | SNP | indel |  | SNP | indel |  |
| Os11g0594400 | Cyclin-like F-box domain containing protein. | 22,639,343 | 22,641,012 | 4 | 2 | 0 |  | 0 | 0 |  | 1 | 0 |  | 0 | 0 | Godinez-Palma et al. 2013 |
| Os11g0594600 | Similar to F-box domain containing protein. | 22,645,772 | 22,647,144 | 8 | 5 | 0 |  | 0 | 0 |  | 2 | 0 |  | 1 | 0 | Godinez-Palma et al. 2013 |
| Os11g0598800 | Tetratricopeptide-like helical domain containing protein. | 22,876,403 | 22,884,442 | 8 | 5 | 0 |  | 4 | 1 |  | 0 | 0 |  | 1 | 0 | Izhaki et al. 2001 |
| Os11g0598850 | Non-protein coding transcript. | 22,886,676 | 22,886,759 | 1^******^ | 0 | 0 |  | 0 | 0 |  | 0 | 0 |  | 0 | 0 | Wu et al. 2019 |
| Os11g0599200 | UDP-glucuronosyl/UDP-glucosyltransferase family protein. | 22,926,178 | 22,928,035 | 1 | 3 | 0 |  | 0 | 0 |  | 0 | 0 |  | 4 | 0 | Liu et al. 2015 |
| Os11g0600500 | Calcium-binding EF-hand domain containing protein. | 23,060,525 | 23,061,100 | 11 | 4 | 0 |  | 0 | 0 |  | 0 | 0 |  | 0 | 0 | Delk et al. 2005 |
| Os11g0601700 | Helix-loop-helix DNA-binding domain containing protein. | 23,132,812 | 23,137,537 | 2 | 0 | 0 |  | 11 | 1 |  | 0 | 0 |  | 1 | 0 | Chen et al. 2018 |

Table S4. continued.

| Gene ID | Description | Start (bp) | End (bp) | CDS^*^ | | |  | Intron | |  | 5' UTR^****^ | |  | 3' UTR | | Related literature^*****^ |
| --- | --- | --- | --- | --- | --- | --- | --- | --- | --- | --- | --- | --- | --- | --- | --- | --- |
|  |  |  |  | nsSNP^**^ | sySNP^***^ | indel |  | SNP | indel |  | SNP | indel |  | SNP | indel |  |
| Os11g0602300 | Similar to HVA22-like protein a (AtHVA22a). | 23,171,943 | 23,176,340 | 1 | 2 | 0 |  | 2 | 0 |  | 1 | 0 |  | 0 | 0 | Guo anf Ho. 2008 |
| Os11g0605800 | Non-protein coding transcript. | 23,375,169 | 23,376,295 | 1 | 0 | 0 |  | 0 | 0 |  | 0 | 0 |  | 0 | 0 | Wu et al. 2019 |
| Os11g0607100 | Pentatricopeptide repeat domain containing protein. | 23,427,261 | 23,429,925 | 3 | 4 | 0 |  | 1 | 0 |  | 0 | 0 |  | 1 | 0 | Sechet et al. 2015 |
| Os11g0609600 | Similar to 14-3-3-like protein. | 23,553,177 | 23,558,494 | 5 | 3 | 0 |  | 43 | 3 |  | 1 | 0 |  | 25 | 2 | Schoonheim et al. 2007 |
| Os11g0610600 | Zinc finger, RING-type domain containing protein. | 23,610,705 | 23,612,241 | 5 | 2 | 0 |  | 0 | 0 |  | 0 | 0 |  | 0 | 0 | Zhang et al. 2015 |
| Os11g0610700 | WD40 repeat-like domain containing protein. | 23,615,076 | 23,619,274 | 4 | 3 | 0 |  | 41 | 4 |  | 1 | 0 |  | 8 | 4 | Gao et al. 2018 |
| Os11g0622700 | Non-protein coding transcript. | 24,302,517 | 24,307,849 | 5 | 0 | 0 |  | 45 | 3 |  | 0 | 0 |  | 0 | 0 | Wu et al. 2019 |
| Os11g0622800 | Putative cinnamyl alcohol dehydrogenase 4. | 24302518 | 24,307,835 | 2 |  | 0 |  | 48 | 0 |  | 0 | 0 |  | 0 | 0 | Zhuang et al. 2013 |

Table S4. continued.

| Gene ID | Description | Start (bp) | End (bp) | CDS^*^ | | |  | Intron | |  | 5' UTR^****^ | |  | 3' UTR | | Related literature^*****^ |
| --- | --- | --- | --- | --- | --- | --- | --- | --- | --- | --- | --- | --- | --- | --- | --- | --- |
|  |  |  |  | nsSNP^**^ | sySNP^***^ | indel |  | SNP | indel |  | SNP | indel |  | SNP | indel |  |
| Os11g0628101 | Non-protein coding transcript. | 24,628,996 | 24,629,376 | 1 | 0 | 0 |  | 0 | 0 |  | 0 | 0 |  | 0 | 0 | Wu et al. 2019 |
| Os11g0631800 | Non-protein coding transcript. | 24,824,252 | 24,825,165 | 1 | 0 | 0 |  | 0 | 0 |  | 0 | 0 |  | 0 | 0 | Wu et al. 2019 |
| Os11g0634350 | Non-protein coding transcript. | 24,958,875 | 24,960,194 | 1 | 0 | 0 |  | 0 | 0 |  | 0 | 0 |  | 0 | 0 | Wu et al. 2019 |
| Os11g0634700 | Similar to Helix-loop-helix DNA-binding domain containing protein. | 24,980,227 | 24,984,249 | 3 | 1 | 0 |  | 7 | 1 |  | 0 | 0 |  | 0 | 0 | Chen et al. 2018 |
| Os11g0634902 | Non-protein coding transcript. | 24,989,149 | 24,993,465 | 1 | 0 | 0 |  | 0 | 0 |  | 0 | 0 |  | 0 | 0 | Wu et al. 2019 |

**^*^**CDS, coding sequence; **^**^**nsSNP, non-synonymous single nucleotide polymorphism; ^***^sySNP, synonymous SNP; ^****^UTR, untranslated region; ^*****^Literature related with gene description and the terms “seed germination” or “seed dormancy” or “pre-harvest sprouting” in the PubMed database (<https://www.ncbi.nlm.nih.gov/pubmed>). ^******^Single nucleotide polymorphisms in the exons of non-protein coding transcript genes were classified as nsSNPs.

References

Bhatnagar N, Min MK, Choi EH, Kim N, Moon SJ, Yoon I, Kwon T, Jung KH, Kim BG (2017) The protein phosphatase 2C clade A protein OsPP2C51 positively regulates seed germination by directly inactivating OsbZIP10. Plant. Mol. Biol. 93(4-5):389-401

Chen HC, Cheng WH, Hong CY, Chang YS, Chang MC (2018) The transcription factor OsbHLH035 mediates seed germination and enables seedling recovery from salt stress through ABA-dependent and ABA-independent pathways, respectively. Rice 11(1):50. doi: 10.1186/s12284-018-0244-z

Delk NA, Johnson KA, Chowdhury NI, Braam J (2005) CML24, regulated in expression by diverse stimuli, encodes a potential Ca^2+^ sensor that functions in responses to abscisic acid, daylength, and ion stress. Plant Physiology 139(1):240-253

El-Maarouf-Bouteau H, Sajjad Y, Bazin J, Langlade N, Cristescu SM, Balzergue S, Baudouin E, Bailly C (2015) Reactive oxygen species, abscisic acid and ethylene interact to regulate sunflower seed germination. Plant Cell Environ. 38(2):364-74

Fang J, Chai C, Qian Q, Li C, Tang J, Sun L, Huang Z, Guo X, Sun C, Liu M, Zhang Y, Lu Q, Wang Y, Lu C, Han B, Chen F, Cheng Z, Chu C (2008) Mutations of genes in synthesis of the carotenoid precursors of ABA lead to pre-harvest sprouting and photo-oxidation in rice. Plant J. 54(2):177-89

Fedi F, O'Neill CM, Menard G, Trick M, Dechirico S, Corbineau F, Bailly C, Eastmond PJ, Penfield S (2017) Awake1, an ABC-Type transporter, reveals an essential role for suberin in the control of seed dormancy. Plant Physiology 174(1):276-283

Gao Y, Liu J, Chen Y, Tang H, Wang Y, He Y, Ou Y, Sun X, Wang S, Yao Y (2018) Tomato SlAN11 regulates flavonoid biosynthesis and seed dormancy by interaction with bHLH proteins but not with MYB proteins. Hortic. Res. 5:27 doi: 10.1038/s41438-018-0032-3

Godinez-Palma SK, Garcia E, Sanchez MD, Rosas F, Vazquez-Ramos JM (2013) Complexes of D-type cyclins with CDKs during maize germination. J. Exp. Bot. 64(18):5661-5671

Guo WJ, Ho TH (2008) An abscisic acid-induced protein, HVA22, inhibits gibberellin-mediated programmed cell death in cereal aleurone cells. Plant Physiology 148(2):1182-1182

Izhaki A, Swain SM, Tseng TS, Borochov A, Olszewski NE, Weiss D (2001) The role of SPY and its TPR domain in the regulation of gibberellin action throughout the life cycle of Petunia hybrida plants. Plant J. 28(2):181-90

Kirkensgaard KG, Hagglund P, Shahpiri A, Finnie C, Henriksen A, Svensson B (2014) A novel twist on molecular interactions between thioredoxin and nicotinamide adenine dinucleotide phosphate-dependent thioredoxin reductase. Proteins 82(4):607-19

Liu Z, Yan JP, Li DK, Luo Q, Yan Q, Liu ZB, Ye LM, Wang JM, Li XF, Yang Y (2015) UDP-Glucosyltransferase71C5, a major glucosyltransferase, mediates abscisic acid homeostasis in arabidopsis. Plant Physiology 167(4):1659-1670

Majee M, Kumar S, Kathare PK, Wu SQ, Gingerich D, Nayak NR, Salaita L, Dinkins R, Martin K, Goodin M, Dirk LMA, Lloyd TD, Zhu L, Chappell J, Hunt AG, Vierstra R, Huq E, Downie AB (2018) KELCH F-BOX protein positively influences Arabidopsis seed germination by targeting PHYTOCHROME-INTERACTING FACTOR1. PNAS 115:E4120-E4129

Ortiz-Espin A, Iglesias-Fernandez R, Calderon A, Carbonero P, Sevilla F, Jimenez A (2017) Mitochondrial AtTrxo1 is transcriptionally regulated by AtbZIP9 and AtAZF2 and affects seed germination under saline conditions. J. Exp. Bot. 68(5):1025-1038

Schoonheim PJ, Sinnige MP, Casaretto JA, Veiga H, Bunney TD, Quatrano RS, de Boer AH (2007) 14-3-3 adaptor proteins are intermediates in ABA signal transduction during barley seed germination. Plant J. 49(2):289-301

Sechet J, Roux C, Plessis A, Effroy D, Frey A, Perreau F, Biniek C, Krieger-Liszkay A, Macherel D, North HM, Mireau H, Marion-Poll A (2015) The ABA-deficiency suppressor locus HAS2 encodes the PPR protein LOI1/MEF11 involved in mitochondrial RNA editing. Mol. Plant 8(4):644-656

Wu J, Liu CX, Liu ZG, Li S, Li DD, Liu SY, Huang XQ, Liu SK, Yukawa Y (2019) Pol III-dependent cabbage BoNR8 long ncRNA affects seed germination and growth in arabidopsis. Plant Cell Physiol. 60(2): 421-435

Zhang HW, Cui F, Wu YR, Lou LJ, Liu LJ, Tian MM, Ning Y, Shu K, Tang SY, Xie Q (2015) The RING Finger Ubiquitin E3 Ligase SDIR1 targets SDIR1-INTERACTING PROTEIN1 for degradation to modulate the salt stress response and ABA signaling in arabidopsis. Plant Cell 27(1):214-227

Zhou X, Hao H, Zhang Y, Bai Y, Zhu W, Qin Y, Yuan F, Zhao F, Wang M, Hu J, Xu H, Guo A, Zhao H, Zhao Y, Cao C, Yang Y, Schumaker KS, Guo Y, Xie CG (2015) SOS2-LIKE PROTEIN KINASE5, an SNF1-RELATED PROTEIN KINASE3-Type protein kinase, is important for abscisic acid responses in arabidopsis through phosphorylation of ABSCISIC ACID-INSENSITIVE5. Plant Physiology 168(2):659-76

Zhou YP, Wu JH, Xiao WH, Chen W, Chen QH, Fan T, Xie CP. Tian CE (2018) Arabidopsis IQM4, a novel calmodulin-binding protein, is involved with seed dormancy and germination in arabidopsis. Front. Plant Sci. 9:721. doi: 10.3389/fpls.2018.00721

Zhuang W-B , Shi T, Gao Z-H, Zhang Z, Zhang J-Y (2013) Differential Expression of Proteins Associated With Seasonal Bud Dormancy at Four Critical Stages in Japanese Apricot. Plant. Biol. (Stuttg) 15 (1), 233-42
